# The Mammalian Target of Rapamycin and DNA methyltransferase 1 axis mediates vascular endothelial dysfunction in response to disturbed flow

**DOI:** 10.1038/s41598-017-15387-5

**Published:** 2017-11-08

**Authors:** Yun-Peng Zhang, Yi-Tao Huang, Tse-Shun Huang, Wei Pang, Juan-Juan Zhu, Yue-Feng Liu, Run-Ze Tang, Chuan-Rong Zhao, Wei-Juan Yao, Yi-Shuan Li, Shu Chien, Jing Zhou

**Affiliations:** 1 Department of Physiology and Pathophysiology, School of Basic Medical Sciences, Peking University, Key Laboratory of Molecular Cardiovascular Science, Ministry of Education, Beijing, 100191 P.R. China; 2 Department of Bioengineering and Institute of Engineering in Medicine, University of California, San Diego, La Jolla, CA 92093 USA

## Abstract

The earliest atherosclerotic lesions preferentially develop in arterial regions experienced disturbed blood flow, which induces endothelial expression of pro-atherogenic genes and the subsequent endothelial dysfunction. Our previous study has demonstrated an up-regulation of DNA methyltransferase 1 (DNMT1) and a global hypermethylation in vascular endothelium subjected to disturbed flow. Here, we determined that DNMT1-specific inhibition in arterial wall ameliorates the disturbed flow-induced atherosclerosis through, at least in part, targeting cell cycle regulator cyclin A and connective tissue growth factor (CTGF). We identified the signaling pathways mediating the flow-induction of DNMT1. Inhibition of the mammalian target of rapamycin (mTOR) suppressed the DNMT1 up-regulation both *in vitro* and *in vivo*. Together, our results demonstrate that disturbed flow influences endothelial function and induces atherosclerosis in an mTOR/DNMT1-dependent manner. The conclusions obtained from this study might facilitate further evaluation of the epigenetic regulation of endothelial function during the pathological development of atherosclerosis and offer novel prevention and therapeutic targets of this disease.

## Introduction

The earliest atherosclerotic lesions typically originate in a nonrandom pattern, developing preferentially at arterial branches and curvatures, where unstable disturbed blood flow is prevalent^1^. Shear stress resulting from disturbed flow is low and oscillates back and forth. Disturbed flow induces a dysfunctional endothelial cell (EC) phenotype that initiates and perpetuates pro-atherogenesis^1,2^. In contrast, the straight parts of the arteries are protected by pulsatile unidirectional laminar flow, and are therefore relatively atheroresistant. Vascular ECs in these regions are exposed to laminar and pulsatile shear stress that induces expressions of atheroprotective genes in the cells^1,2^. Emerging evidence from our laboratory and others has revealed that epigenetic mechanisms, including DNA methylation, histone modifications, and noncoding RNA-based post-transcriptional regulations play critical roles in the flow-regulation of endothelial gene expression and phenotype^3–9^. Of these, DNA methyltransferase (DNMT) -1 and -3A were reported to be up-regulated by disturbed flow to promote a proatherogenic phenotype in vascular ECs^3,4,8^. However, the signaling pathways through which disturbed flow up-regulates DNMTs are unclear. The mechanism by which DNMTs cause changes in EC gene expression and function is still lacking.

Mapping of the mechanotransduction networks that can be activated by shear stress in vascular ECs has indicated a series of signaling pathways as well as their critical nodes, including integrins^10,11^, the mitogen-activated protein kinases (MAPKs)^12,13^, and the mammalian target of rapamycin (mTOR)^14,15^. By examining ECs in a parallel-plate flow channels exposing to pulsatile shear (PS) that mimics atheroprotective flow or oscillatory shear (OS) that mimics atherogenic flow, our previous studies demonstrated that OS induces sustained phosphorylation of mTOR and its effector ribosomal protein S6 kinase (p70S6K)^14^. Integrins, whose activation induces their associations with focal adhesion kinase (FAK) and Shc, an adaptor protein containing a C-terminal Src homology domain-2 (SH2) domain, and activation of downstream MAPK ERK1/2, have been indicated as mechanosensors in the flow-activation of phosphatidylinositol 3-kinase (PI3K)/mTOR/p70S6K^14,16,17^. There is evidence that the PI3K/mTOR/p70S6K pathway is required for EC proliferation, survival, and migration^18,19^. Interestingly, treatment of ECs with 5-Aza-2′-deoxycytidine (5-Aza), an inhibitor of DNMTs, limited cell proliferation and migration^20,21^. On the basis of these studies, it is speculated that mTOR might mediate the OS-induction of DNMT1, which would lead to downstream changes in gene expression to result in EC dysfunction. Although Shc and FAK have been shown to be critical for the integrin-mediated signaling, whether integrins/Shc/FAK modulate the activation of the putative mTOR-DNMT1 axis in response to OS remains to be determined.

The atherosusceptible endothelium is characterized by elevated expression of proatherogenic genes as well as consequential aberrant proliferation, migration, and pro-inflammatory responses^1^. The proatherogenic genes that can be induced by application of disturbed flow or OS to ECs include cyclin A (CCNA2)^22,23^ and connective tissue growth factor (CTGF)^10,24^, those have been found to be highly expressed in atherosclerotic plaques^25–27^. Cyclin A promotes both G1/S and G2/M transitions of the cell cycle in somatic cells. CTGF has been associated with proliferation and inflammation in ECs and it enhances the chemosensory adhesion and migration of monocytes into atherosclerotic lesions^26,27^. Promoter DNA methylation contributes to the regulatory mechanisms of cyclin A and CTGF by various stimuli such as homocysteine^21^ or high glucose^28^. In particular, a positive correlation of cyclin A promoter methylation with cyclin A expression is found^21^, whereas CTGF promoter methylation is negatively correlated with the CTGF protein level^28,29^. In this study we have examined the role of DNMT1-dependent promoter methylation in the flow-regulation of EC cyclin A and CTGF expression as well as the functional consequences. We also discovered the mechanotransductive mechanisms of OS-specific up-regulation of DNMT1.

## Results

### Silencing of DNMT1 ameliorates the OS-induced endothelial dysfunction

**Figure 1:**
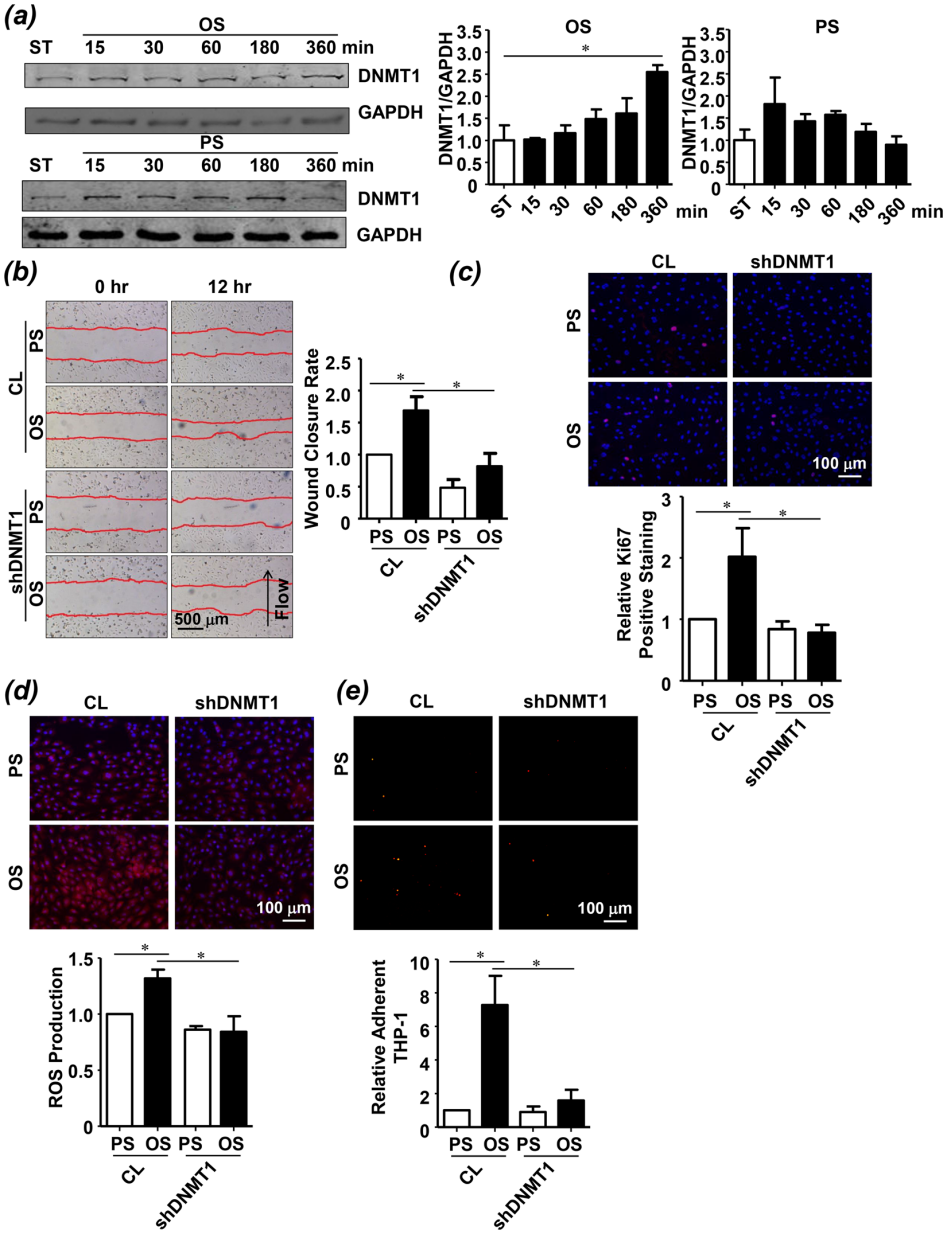
DNMT1-specific inhibition ameliorates the oscillatory shear (OS)-induced endothelial dysfunction. (**a**) ECs were exposed to pulsatile shear PS(12 ± 4 dynes/cm^2^) or OS (0.5 ± 4 dynes/cm^2^) for indicated time, and expression of DNMT1 was analyzed by Western blot. (**b**–**e**) ECs were infected with the control adenovirus (CL) or adenovirus expressing shRNA targeting human DNMT1 (shDNMT1). The infected ECs were exposed to PS or OS for 6 hours, cell migration was assessed by scratch wound healing assay (**b**), cell proliferation was indicated by immunofluorescent staining of the proliferative marker, Ki67 (**c**), ROS production was assessed (**d**), and monocyte adhesion assay was performed to exam the cellular inflammatory response. Black arrow indicates the flow direction. Images are representative of 3–5 independent experiments with similar results. *P < 0.05 compared with the indicated controls.

Our previous study demonstrated that atheroprone OS induces DNA hypermethylation and DNMT1 expression in comparison to atheroprotective PS after 24 hours of shearing^8^. To investigate the temporal regulation of endothelial DNMT1 expression by different patterns of shear stress, OS at 0.5 ± 4 dyn/cm^2^ or PS at 12 ± 4 dyn/cm^2^ was applied over a time course of 6 hours to human umbilical vein ECs (HUVECs). Expression of DNMT1 was induced gradually by OS and remained elevated after 6 hours of shearing, compared with the control cells under static condition (Fig. 1a). This induction lasted over 24 hours of OS application (Fig. S1). In contrast, a rapid induction of DNMT1 expression was observed in ECs exposed to PS; however, the induction was transient and returned to the basal level after 6 hours of shearing (Figs 1a and S1). The followed functional experiments were therefore conducted after 6 hours of shearing unless otherwise specified. Because treatment of static ECs with non-specific DNMT inhibitor 5-Aza suppresses migration and proliferation of the cells, we explored the mediatory role of DNMT1 in OS-induced endothelial migration and proliferation by silencing DNMT1 with recombinant adenovirus expressing shRNA targeting human DNMT1 (ad-*h*shDNMT1). As evidenced by scratched wound healing assay and immunofluorescent staining of proliferative marker Ki67, exposure of ECs to OS accelerated cell migration and promoted aberrant cell proliferation; these effects were abolished by DNMT1 silencing (Fig. 1b,c). These observations suggest a pro-migrative and pro-proliferative role of DNMT1 in mediating the OS-induced endothelial dysfunction. Production of reactive oxygen species (ROS) and adhesion of circulating monocyte to endothelium have been associated with endothelial inflammation. We found an increased ROS production and an enhanced THP-1 monocyte adhesion in ECs exposed to OS (Fig. 1d,e). Silencing of DNMT1 suppressed the OS-induced production of ROS and decreased the relative amount of THP-1 monocytes adherent to the endothelial monolayer (Fig. 1d,e). Together, these results indicated that DNMT1 mediates the functional modulation of OS on ECs *in vitro*.

### DNMT1 expression and global DNA methylation are elevated in the atherosclerotic endothelium

**Figure 2:**
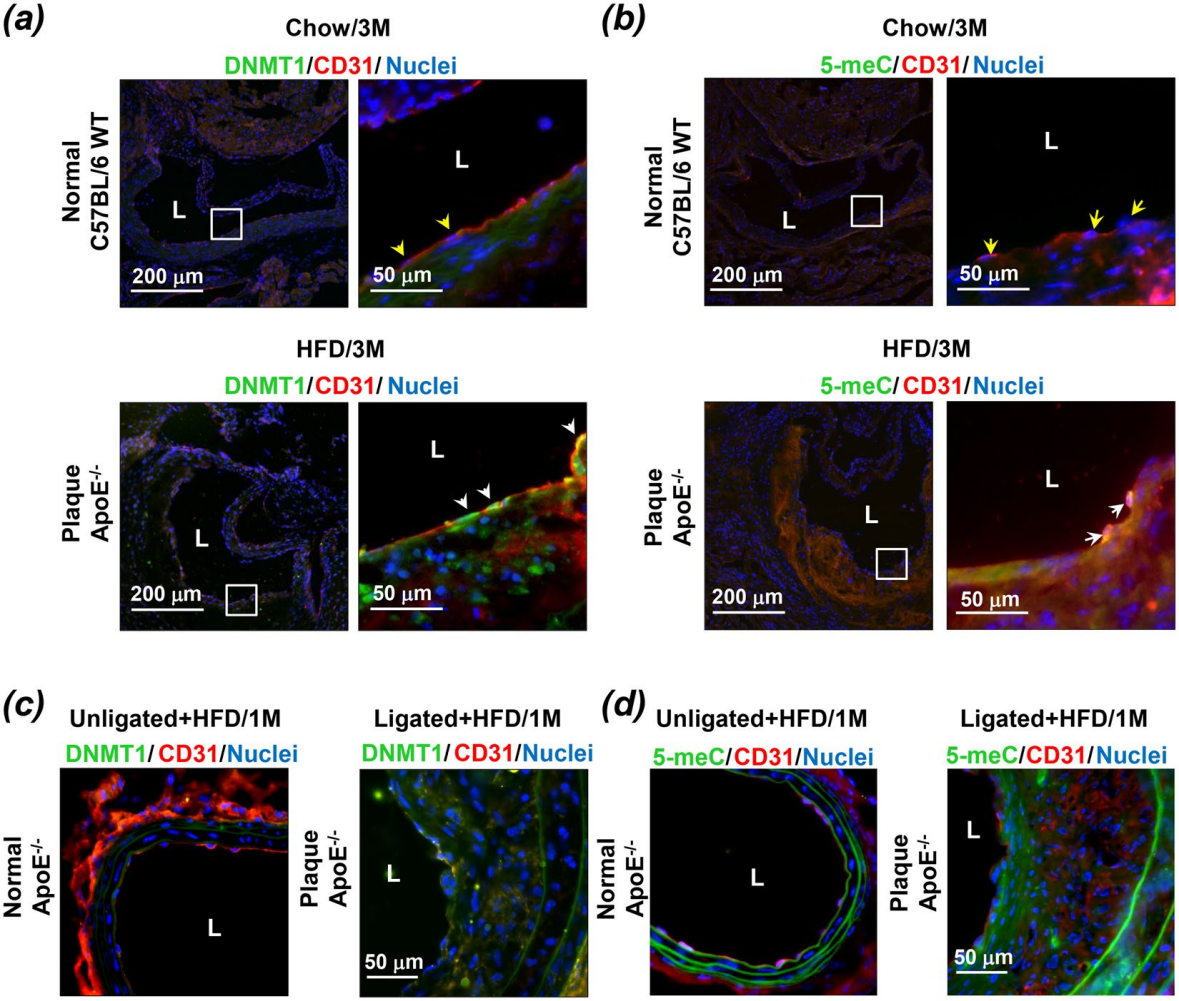
DNMT1 expression and global DNA methylation are elevated in the atherosclerotic endothelium in mice model. (**a**,**b**) Expressions of DNMT1, endothelial marker CD31 and methylated 5-cytosine (5-meC) were measured by immunofluorescent staining in aortic outflow from C57BL/6 WT mice with Chow diet (upper panels, yellow arrow heads indicate ECs without DNMT1 or 5-meC expressions) and Apolipoprotein E-deficient (ApoE^−/−^) mice fed a Western diet (lower panels, white arrow heads indicate ECs expressing high level of DNMT1 expression or 5-meC). (**c**,**d**) Expressions of DNMT1, endothelial marker CD31, and methylated 5-cytosine (5-meC) were measured by immunofluorescent staining in the unligated carotid arteries (left panels) and the ligated carotid arteries (right panels) from ApoE^−/−^ mice fed a Western diet. L: lumen; 1 M: one month; 3 M: three months. Images are representative of 8–10 mice with similar results.

To gain insight into the *in vivo* relevance and functional implications of DNMT1 in atherosclerosis, we examined DNMT1 expression and global methylation of its substrate, DNA cytosine-5, in atherosclerotic plaques. Apolipoprotein E-deficient (ApoE^−/−^) mice fed a high-fat diet (HFD) for three months and the control C57BL/6 wild-type (WT) mice were euthanized and DNMT1 and methylated 5-cytosine (5-meC) were examined in the aortic roots. CD31 was co-stained to indicate the EC layer. The control aorta from WT mice showed weak signals of DNMT1 and 5-meC in the arterial walls (Fig. 2a,b, upper panels; Fig. S2a,b). In contrast, a pronounced staining of DNMT1 and 5-meC was observed both in the EC layers and in the plaques (Fig. 2a,b, lower panels; Fig. S2a,b). These findings reveal a high prevalence of DNMT1 expression and DNA hypermethylation in the atherosclerotic lesions, rather than in the undiseased regions.

To explore the involvement of DNMT1 in disturbed flow-induced endothelial dysregulation as well as atherogenesis, we studied DNMT1 expression and DNA methylation in endothelium in a mouse carotid partial ligation model, in which 3 of the 4 caudal branches of the left common carotid artery are surgically ligated (ligated), while the contralateral right common carotid artery remains untouched and serves as control (unligated). Flow disturbance was therefore generated in the left common carotid arteries. Partial ligation in ApoE^−/−^ mice fed a HFD resulted in atheroma formation in one month (Fig. 2c,d; Fig. S2c,d). Consistent with observations from the three-month-HFD model, the flow-disturbance-accelerated atherosclerosis model showed up-regulated DNMT1 expression and DNA methylation in the EC layers and in the lesion regions, in comparison with those in the normal regions (Fig. 2c,d; Fig. S2c,d). Altogether, the strong correlation between DNMT1 expression and atherosclerosis suggests a positive contribution of DNMT1 to atherogenesis.

### Inhibition of DNMT1 ameliorates endothelial dysfunction and atherosclerosis

**Figure 3:**
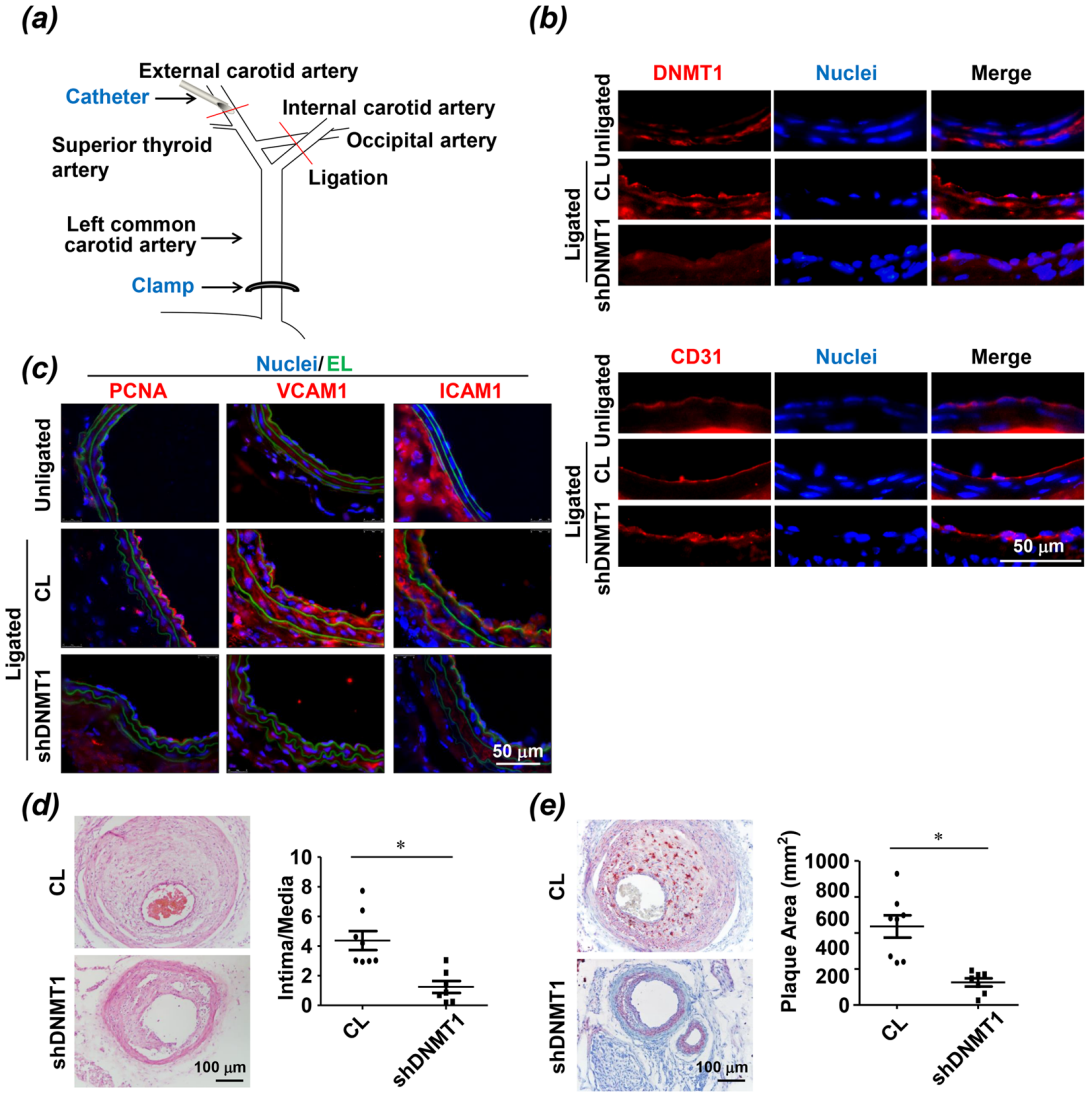
Intraluminal inhibition of DNMT1 ameliorates endothelial dysfunction and atherosclerosis. (**a**) Schematic diagram of local delivery of virus. (**b**) At one week post-surgery, the DNMT1-knockdown efficiency in the ligated carotid arteries was validated by immunofluorescent staining. CD31 indicates the intact endothelia. (**c**) Cell proliferation marker PCNA (left panels) and inflammatory markers VCAM1 and ICAM1 (middle and right panels) were assessed by immunofluorescence staining in the unligated and ligated carotid arteries subjected to a locally intraluminal incubation with adenovirus expressing shRNA targeting murine DNMT1 (shDNMT1) or control virus. (CL). EL: elastic lamina. (**d**,**e**) At four weeks post-surgery, neointimal formation and atherosclerosis were analyzed by haematoxylin and eosin (H&E) and Oil red O stainings in the ligated carotid arteries from ApoE-deficient mice fed a Western diet. The mouse carotid arteries were subjected to a locally intraluminal incubation with ad-*m*shDNMT1 or control virus. Images are representative of 8–10 mice with similar results. *P < 0.05 vs. CL.

We next assessed the functional consequence of DNMT1-specific silencing in the partial ligation model. For knockdown of vascular DNMT1, the mice received a locally intraluminal incubation of recombinant adenovirus expressing GFP and shRNA targeting murine DNMT1 (ad-*m*shDNMT1) (Fig. 3a). Intraluminal incubation of ad-*m*shDNMT1 but not the control virus expressing GFP decreased the arterial expression of DNMT1, as evidenced by immunofluorescent staining performed 7 days after the operation (Fig. 3b, upper panels). Staining of CD31 indicated an intact or regenerated endothelium upon virus incubation (Fig. 3b, lower panels). An increased expression of DNMT1 in the endothelium of partially ligated arteries compared with that in the unligated was observed (Fig. 3b, upper panels). Immunofluorescence also indicated prominent expressions of proliferating cell nuclear antigen (PCNA), vascular cell adhesion molecule 1 (VCAM1), and intercellular cell adhesion molecule 1 (ICAM1), in the vessel walls of the ligated carotid arteries with control virus, in comparison with those in the right carotid arteries without operation and with those subjected to ad-*m*shDNMT1 incubation (Fig. 3c). Nuclear expression of PCNA reveals cell proliferation while the endothelial adhesion molecules VCAM1 and ICAM1 facilitate the accumulation of circulating monocytes on the endothelium. These results suggest that DNMT1 promotes endothelial proliferation and inflammation *in vivo*.

Moreover, we investigated the effects of DNMT1 silencing by ad-*m*shDNMT1 on atheroma formation. The ligated arteries with intraluminal incubation of control virus rapidly developed atherosclerosis within 4 weeks of operation, and this was markedly attenuated in the arteries with incubation of ad-*m*shDNMT1, as quantified by intima-to-media ratio and plaque area (Fig. 3d,e). Taken together, these findings provide strong evidence that DNMT1 silencing prevents atherosclerosis by reducing endothelial proliferation and pro-inflammation in the arterial wall.

### mTOR/p70S6K mediates the OS-induction of DNMT1

**Figure 4:**
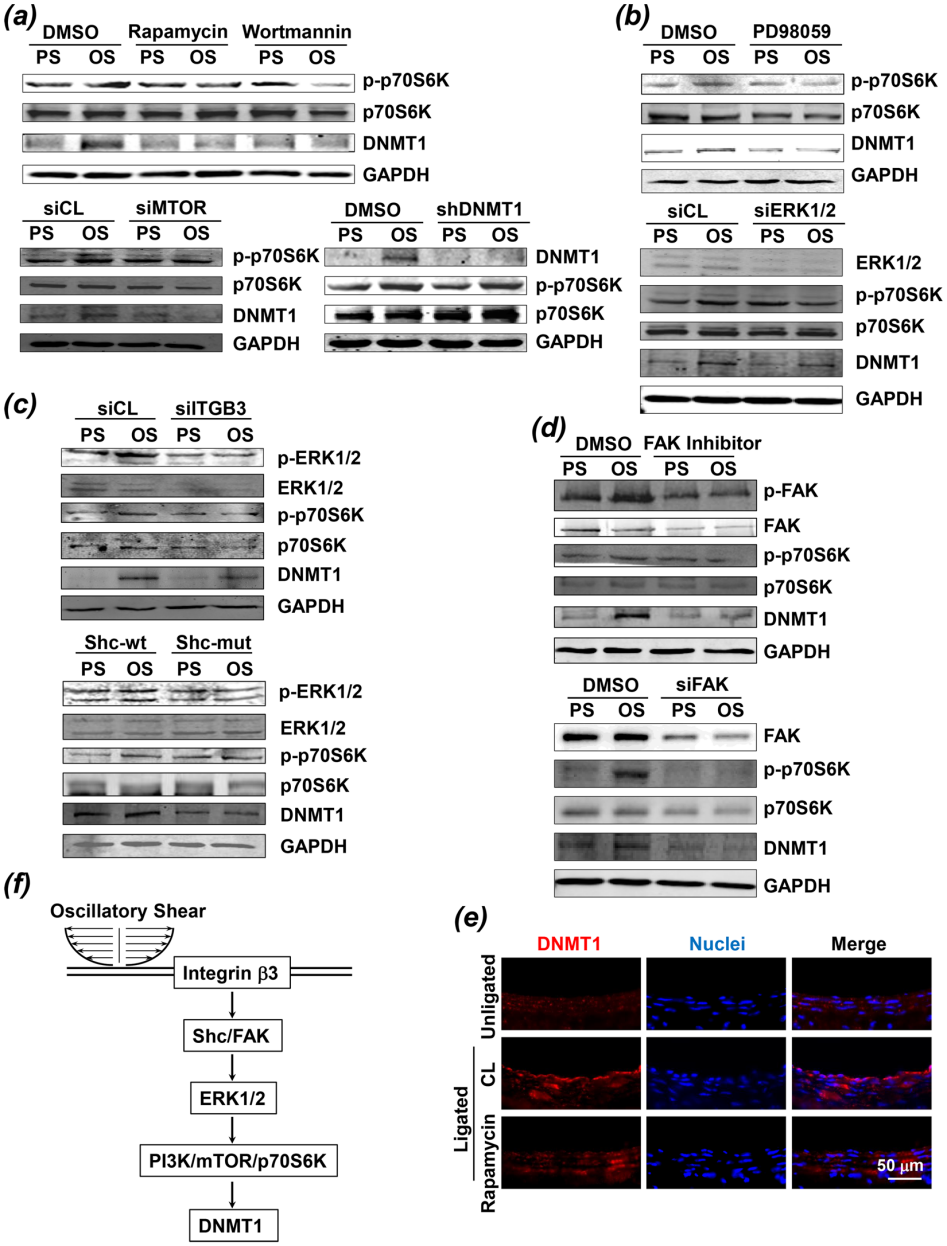
OS-induction of DNMT1 is dependent on the integrin/Shc/FAK/ERK/mTOR/p70S6K signaling pathways. (**a**–**d**) ECs were incubated with rapamycin (50 nM), wortmannin (100 nM) *(a, upper panels)*, PD98059 (50 nM) *(b)*, FAK inhibitor (50 nM) (**d**), or infected with adenovirus expressing shRNA targeting human DNMT1 (shDNMT1) *(a, lower panels)*, or transfected with siRNAs targeting mTOR, ERK1/2, FAK, or integrin β3 *(a, lower panels; b, lower panels; c, upper panels; d, lower panels)*, or transfected with the wild-type (Shc-wt) or mutant (Shc-mut) of Shc constructs *(c, lower panels)*. The pretreated cells were exposed to PS and OS for 6 hours and were then subjected to Western blot. Images are representative of 3 independent experiments with similar results. (**e**) Immunofluorescent-staining of DNMT1 in unligated or ligated carotid arteries from C57B/L6 wild-type (WT) mice. The mice were subjected to intraperitoneal injection with rapamycin every other day (1 mg/kg body weight) or control vehicle. Images are representative of 8–10 mice with similar results. (**f**) Schematic diagram of signaling pathways that mediate the OS-induction of DNMT1.

We investigated the molecular mechanisms underlying the OS-induction of DNMT1. ECs were pre-incubated with mTORC1 inhibitor, rapamycin, at 50 nM for 12 hours after seeding onto the collagen-coated glass slides, and then exposed to either PS or OS for 6 hours. Rapamycin blocked the OS-induced phosphorylation of mTOR’s effector, p70S6K, as well as the OS-induction of DNMT1 expression, as compared with the cells pretreated with control reagent, DMSO (Fig. 4a, upper panels). Pre-treating ECs with wortmannin (100 nM, 12 hours), which is known to target PI3K/mTOR cascade upstream of p70S6K, also suppressed the OS-induction of DNMT1 (Fig. 4a, upper panels). Coinciding with these results from experiments of pharmacological inhibition, knockdown of mTOR expression by siRNA silencing blocked the DNMT1 up-regulation (Fig. 4a, lower left panels). Inhibition of DNMT1 by ad-*h*shDNMT1 did not affect the OS-activation of p70S6K, suggesting no feedback control of mTOR/p70S6K signaling by DNMT1 (Fig. 4a, lower right panels). In our previous study we found that application of OS to ECs induced ERK1/2 MAPK phosphorylation, which directed the activation of mTOR^14^. Therefore we tested the involvement of ERK1/2 activation in the shear stress-regulation of DNMT1. We observed that the OS-induced p70S6K phosphorylation and DNMT1 expression were inhibited by pretreating ECs with PD98059 (50 nM), an inhibitor of MAPK kinase (MEK) that is upstream of ERK, or by transfecting the cells with siRNAs targeting ERK1 and ERK2 (Fig. 4b).

To explore the mechanosensors that perceive shear stress and translate this stimulus into biochemical signals to induce DNMT1 expression, ECs were transfected with siRNA targeting integrin β3 (siITGB3, 40 nM) or the control scrambled siRNA (siCL), and then exposed to either PS or OS. Knockdown of integrin β3 inhibited the OS-induced phosphorylation of ERK1/2 and p70S6K, and expression of DNMT1 (Fig. 4c, upper panels). Transfecting ECs with a dominant-negative mutant of Shc (Shc-mut) but not with the wild typo control (Shc-wt) also resulted in inhibitions in OS-induced ERK1/2 and p70S6K phosphorylation as well as DNMT1 expression (Fig. 4c, lower panels), confirming the involvement of integrin and its adaptor protein, Shc, in mediating the shear-initiated ERK/mTOR/DNMT1 signaling. Integrin-regulated protein focal adhesion kinase (FAK) has been associated with shear-activation of MAPK. We verified the contribution of FAK in directing the OS-induced mTOR activation and DNMT1 expression by pre-incubating ECs with a FAK inhibitor or the control reagent, DMSO, and then exposed the cells to OS or PS. FAK inhibition suppressed the OS-induction of p70S6K phosphorylation and DNMT1 expression (Fig. 4d, upper panels). Transfection of cells with siRNA targeting FAK also resulted in the suppression of OS-induced DNMT1 expression (Fig. 4d, lower panels).

In summary, these *in vitro* studies demonstrate the importance of integrin-MAPK-mTOR cascade in mediating the shear stress-induction of DNMT1 expression. Evidence from *in vivo* experiments confirmed this signaling mechanism by showing that the endothelial expression of DNMT1 in the ligated left carotid arteries of WT mice was inhibited by repetitive intraperitoneal injection of rapamycin (Fig. 4e). The *in vitro* and *in vivo* studies strongly support a signaling model as summarized in the schematic diagram (Fig. 4f).

### DNMT1 targets cyclin A and CTGF

**Figure 5:**
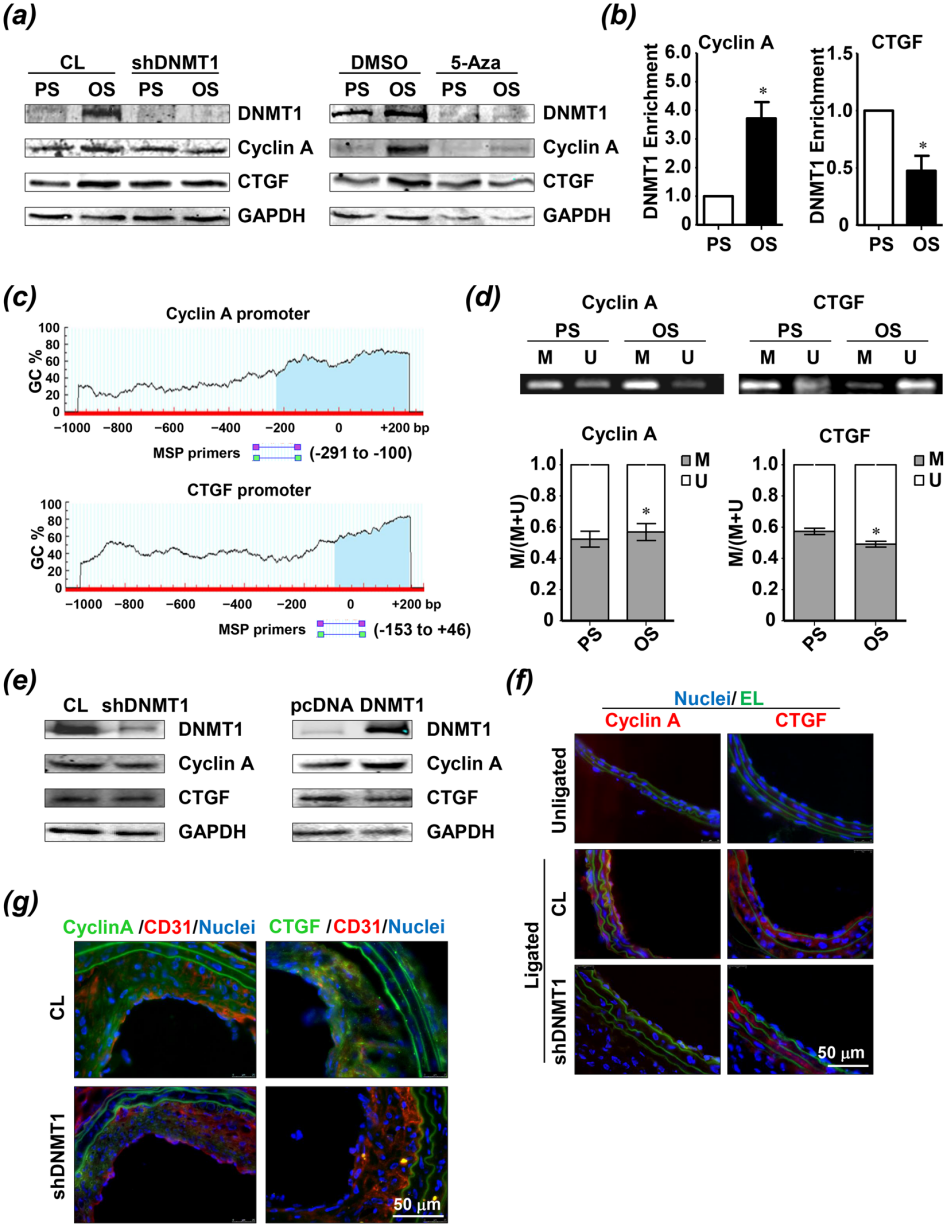
DNMT1 regulates the OS-induced expressions of CTGF and cyclin A via directly targeting their promoters. (**a**) ECs were infected with adenovirus expressing shDNMT1 or treated with DNMT inhibitor 5-Aza-2′-deoxycytidine (5-Aza, exposed to PS or OS for 6 hours, and expressions of DNMT1, cyclin A, and CTGF were assessed by Western blot. (**b**) ECs were exposed to PS or OS for 6 hours. After that, DNMT1 binding at CTGF and cyclin A promoter regions was analyzed by chromatin immunoprecipitation (ChIP) assay. (**c**) CpG island search in the promoter regions of cyclin A and CTGF. The shade shows regions with GC percentage greater than 50%. The locations of the methylation-specific PCR (MSP) primers are illustrated. (**d**) ECs were exposed to PS or OS for 6 hours. After that, the methylation levels of the CTGF and cyclin A promoter were measured by MSP. M: methylated; U: unmethylated. (**e**) ECs were infected with control virus or ad-*h*shDNMT1, or transfected with control plasmid or plasmid overexpressing DNMT1. Expressions of DNMT1, cyclin A, and CTGF were analyzed by Western blot. (**f**) Immunofluorescent-staining of cyclin A and CTGF in unligated and ligated carotid arteries from C57B/L6 wild-type (WT) mice at one week after operation. The ligated carotid arteries were subjected to a locally intraluminal incubation with ad-*m*shDNMT1 or control virus. (**g**) Immunofluorescent-staining of cyclin A and CTGF in unligated and ligated carotid arteries from ApoE^−/−^ mice at four weeks after operation. Images are representative of 3 independent experiments or 8–10 mice with similar results. *P < 0.05 vs. PS.

We then searched for unidentified flow-sensitive genes whose expressions are differentially regulated by OS versus PS in a DNMT1-dependent manner. Candidates for screening included cyclin A, CTGF, endothelial nitric oxide synthase (eNOS), Kruppel-like factor 2 (KLF2), KLF4, and others. Of these, cyclin A and CTGF showed robust up-regulation upon OS exposure, and the up-regulation could be completely prevented by infecting the cells with ad-*h*shDNMT1 or by pretreating the cells with 5-Aza (Fig. 5a). The OS induced cell proliferation and THP-1 adhesion could be suppressed by siRNA-mediated silencing of cyclin A and CTGF, respectively (Fig. S3). Mechanistically, OS increased the binding of DNMT1 to the promoter regions of cyclin A, whereas it reduced DNMT1 binding to the promoter regions of CTGF (Fig. 5b), as evidenced by chromatin immunoprecipitation assay. Because DNMT1 catalyzes DNA primarily at CpG islands within the promoter regions, we analyzed the DNA sequence at the 5′-flanking regions of human cyclin A and CTGF genes using a CpG island search program, and detected CpG-rich regions (GC content > 50%) that span positions −291 to −100, and −153 to +46, around the promoters of the cyclin A and CTGF genes, respectively (Fig. 5c). We used methylation-specific PCR (MSP) analysis to study promoter methylation of cyclin A and CTGF genes. Application of OS to ECs resulted in hypermethylation of cyclin A promoter and hypomethylation of CTGF promoter, in comparison with application of PS to the cells (Fig. 5d). These observations are in line with the shear stress-regulated DNMT1 binding to the cyclin A and CTGF promoters. However, the decrease of DNMT1 in DNA binding ability to CTGF promoter is apparently conflicting with the increase in DNMT1 expression upon OS stimulation. Therefore we next employed gain- and loss-of-function approaches to study the correlation between expressions of DNMT1 and CTGF in static condition. Expression of cyclin A was also measured in parallel experiments. Western blot analysis revealed that cyclin A was down-regulated by DNMT1 inhibition and was up-regulated by DNMT1 overexpression; and that CTGF expression was negatively correlated to the DNMT1 expression (Fig. 5e). Given that OS induced CTGF expression and this induction was dependent on DNMT1 (Fig. 5a), these results implied that overexpressing DNMT1 in static condition solely could not mimic OS.

Targeting of cyclin A and CTGF by DNMT1 was verified *in vivo* by immunofluorescent staining of cyclin A and CTGF in the ligated mouse carotid arteries with or without intraluminal silencing of DNMT1. For both the one-week and four-week models, the ligated arterials had increased levels of cyclin A and CTGF in the vessel wall, in particular the endothelium, in comparison with the unligated (Fig. 5f,g). The increase was suppressed in the arteries with DNMT1 inhibition (Fig. 5f,g).

## Discussion

DNMTs have been shown to play important roles in cardiovascular biology, in particular, the regulation of endothelial lineage and homeostasis^30–32^. Recent studies demonstrated that DNMT1is aberrantly expressed in endothelial cells in response to various stimuli, including atheroprone hemodynamic shear stress^3,8^. Our previous report and present findings also support the pro-atherogenic role of DNMT1 in vascular ECs subjected to low and oscillating shear stress. This study specifies the function of endothelial DNMT1 to be pro-proliferative, pro-migratory, and pro-inflammatory (Fig. 1b–e). The conclusions we obtained *in vitro* were further supported by the *in vivo* results that in diseased arterial walls, DNMT1 was dysregulated and highly expressed (Fig. 2a,c). Accordingly, DNAs from the atherosclerotic plaques were hypermethylated in comparison with those from the plaque-free regions (Fig. 2b,d). Although there is seemingly contradictory evidence in the literature revealing that demethylation of normally hypermethylated CpG islands occurs in human atherosclerotic arteries^33,34^, recent study using genome-wide DNA methylation profiling at single-nucleotide resolution indicated that DNA hypermethylation is as an overlapping feature of human atherosclerosis and an ultimate outcome of atherosclerosis-related stimuli^35^. Explaining this controversy is difficult, because none of these studies considered dissecting the contribution of ECs to hypermethylation or hypomethylation. Our results are in agreement with the notion of “global DNA hypermethylation” in atherosclerotic plaques. In particular, we attribute the gross hypermethylation, at least in part, to the endothelial up-regulation of DNMT1 and endothelial hypermethylation. Moreover, this presented study may facilitate the establishment of a causal relationship between expression of endothelial DNMT1 and the pro-atherogenic endothelial dysfunction and offer opportunities for therapeutic intervention of atherosclerosis. The latter is further supported by the compelling evidence from intraluminal DNMT1 inhibition in the partial ligation model, that silencing of vascular DNMT1 suppressed the disturbed flow-induced expressions of proliferating marker PCNA and inflammatory molecules VCAM1 and ICAM1, and attenuated the flow-disturbance-augmented atherosclerosis (Fig. 3c–e).

The mechanotransduction processes depend on the mode of shearing, thus the mechano-chemical signalings have been studied by comparing the effects of no-shearing, shearing with a significant forward direction (whether laminar shear without oscillation^36^ or PS in which an oscillatory component is superimposed on a steady shearing), and shearing without a significant forward direction or with little net forward component, e.g., OS. In this study we directly compared the effects of atheroprotective PS and atheroprone OS loading on ECs. By comparison, many other studies reported the effects of shear stress with certain flow pattern when referenced to a static condition. However, the no-shearing condition does not exist *in vivo* because of the existence and variation of flow velocity and pressure in the vessels. We also conducted time-course experiments to investigate the flow-regulation of DNMT1 expression within 6 hours of shearing. DNMT1 was transiently induced by PS, by contrast, was gradually and sustainedly induced by OS (Fig. 1a). The transient changes of gene expression upon PS exposure may be caused by shifting instantaneously from static to shear condition. At 6 hours of shearing, the majority of cells have recovered from the adaptive responses and the OS-specific induction of DNMT1 becomes prominent. As summarized in Fig. 4f, we have demonstrated a novel mechanism by which OS specifically up-regulates DNMT1. Our data suggests that OS-induction DNMT1 is dependent on integrin β_3_ (Fig. 4c), whose activation is then transmitted into cells through the phosphorylation of integrin-associated proteins such as Shc and FAK (Fig. 4c,d). Shc and FAK signaling has been shown to be critical for the shear stress-activation of MAPKs^14^. Our results are consistent with the previous findings and indicate that ERK1/2 plays an important role in mediating the activation of mTOR and the subsequence DNMT1 up-regulation (Fig. 4b). The role of mTOR signaling in modulating DNMT1 expression in cells is controversial. The level of DNMT1 was higher after treatment of the hepatocellular carcinoma cell lines with an mTOR inhibitor, Torin-2, whereas the increase in DNMT1expression by TGF-β treatment was abolished in fibroblasts by FAK, PI3K, and mTOR inhibitors, including rapamycin^37,38^. It is possible that the effects of activating mTOR on DNMT1 expression are diverse with respect to the cell types and stimuli. Our findings showed that treatment of ECs with mTOR inhibitor rapamycin or PI3K inhibitor wortmannin blocked the OS-induction of DNMT1 (Fig. 4a), indicating that PI3K/mTOR activations are necessary for DNMT1 up-regulation in response to OS. The mediators between mTOR/p70S6K and DNMT1 warrant further characterizations.

Cyclins associate with cyclin-dependent protein kinases (CDKs) to regulate their activity and the progression of the cell cycle. Cyclin A is highly expressed in atherosclerosis^25^. We have previously demonstrated that OS promotes aberrant EC proliferation and cell cycle progression through up-regulation of cyclin A expression^14^. Research from others indicated that the EC expression of cyclin A could be inhibited by homocysteine through down-regulating DNMT1 and the subsequent hypomethylation of cyclin A promoter^21^. Our current study showed that OS increases DNMT1 to methylate the cyclin A promoter and therefore increases cyclin A expression (Fig. 5), confirming and extending the observations from ours and others. Our findings also support the notion that DNA methylation on promoters can activate gene transcription, probably through the cooperation with other epigenetic machineries such as histone acetylates/deacetylates, histone methyltransferases/demethylases, and chromatin remodeling complexes. In previous reports, pro-atherogenic CTGF has been suggested to be increased by disturbed flow but not by steady laminar flow in ECs^10,24^. Here we identified an epigenetic modulator, DNMT1, which mediates the disturbed flow-induction of CTGF. Similar to the case of cyclin A that is regulated by DNMT1, CTGF expression is positively correlated to DNMT1 expression under shearing conditions, *in vitro* and *in vivo* (Fig. 5a,f and g). However, the positive correlation does not exist in static condition (Fig. 5e). Besides, OS reduced the binding of DNMT1 to CTGF promoter as well as the methylation of CTGF promoter (Fig. 5b,d). One possible explanation for the contradictory observations between the increased expression and decreased site-specific binding of DNMT1 in response to OS may be that some co-factors are required for recruiting or competing with DNMT1 to bind the CTGF promoter. It is likely that OS regulates the co-factors to dominate the DNMT1 binding. Further investigation will be required to characterize the detailed mechanism by which OS induces a site-specific binding and distribution of DNMT1 along the genome.

## Methods

### Cell culture and shear experiments

Human umbilical vein endothelial cells (HUVECs) within passages 5–8 were maintained in Medium 199 (Gibco) supplemented with 10% fetal bovine serum (FBS) (Gibco). Bovine artery endothelial cells (BAECs) within passages 5–18 were maintained in Medium DMEM (Gibco) supplemented with 10% fetal bovine serum (FBS) (Gemini). A parallel-plate flow apparatus was used to impose fluid shear stress to the cells seeded on collagen I (50 μg/mL)-coated glass slides, as described previously^14^. The shear stress (τ) generated on the endothelial monolayer was estimated as 6Qμ/wh^2^, where Q is flow rate and μ is perfusate viscosity. ECs were exposed to either pulsatile shear (PS, 12 ± 4 dynes/cm^2^) or oscillatory shear (OS, 0.5 ± 4 dynes/cm^2^).

### Fluorescent immunocytochemistry

ECs on glass slides were fixed in 4% paraformaldehyde for 15 minutes, permeabilized with cold PBS containing 0.4% Triton X-100 for 10 minutes, and incubated with blocking buffer (3% bovine serum albumin in PBS) for 1 hour before incubation overnight with primary antibodies against 5-methylcytosine (5-meC) (Eurogentec), CD31(Santa Cruz Biotech) or DNMT1 (Santa Cruz Biotech). For 5-meC staining, the permeabilized cells were denatured with 2 N HCl and neutralized with 100 mM Tris-HCl (pH 8.5) before blocking. The cells were washed, incubated with secondary antibodies, and mounted in fluorescent mounting medium with DAPI. The slides/slips were then visualized by epi–fluorescence microscopy.

### Western blot

ECs were lysed in the RIPA lysis buffer: 25 mM HEPES, pH 7.4, 1% Triton X-100, 1% deoxycholate, 0.1% SDS, 125 mM NaCl, 5 mM EDTA, 50 mM NaF, 1 mM PMSF. Equal amounts of protein were separated on SDS-PAGE, transferred to nitrocellulose membranes, blocked with 5% skim milk TBST, and incubated with the primary antibodies against DNMT1 (Santa Cruz Biotech), p-ERK (Bioworld), ERK (Bioworld), p-p70S6K (Santa Cruz Biotech), p70S6K (Proteintech), p-FAK (Santa Cruz Biotech), FAK (Proteintech) or GAPDH (Santa Cruz Biotech).

### Tissue preparation, hematoxylin and Oil red O staining, and immunofluorescence

Frozen blocks containing the hearts, aortic arches, and carotid arteries were prepared in TissueTek and stored at −80 °C. Sections from the blocks were stained with Oil red O by fixing of the frozen tissues with 10% formalin for 10 minutes, rinsing twice with distilled water for 5 minutes, orbital shaking in 60% propanediol for 10 minutes, and incubation in 0.2% Oil red O solution for 5 minutes at room temperature. For hematoxylin staining, the previous method was followed by 2 rinses with distilled water, a hematoxylin dip for 20 seconds, 2 rinses with distilled water and then mounting. Immunofluorescence was performed on sections from the frozen blocks using the following antibodies at a 1:150 dilution: DNMT1 (Santa Cruz Biotech), PCNA (Santa Cruz Biotech), VCAM1 (Santa Cruz Biotech), ICAM1 (Santa Cruz Biotech), cyclin A (Santa Cruz Biotech),CTGF (Santa Cruz Biotech), CD31 (Santa Cruz Biotech).

### Monocyte adhesion assay

ECs were pre-incubated with adenovirus and were subjected to either PS or OS for 6 hours. Human peripheral blood mononuclear leukocytes (THP-1 cells) were cultured in RPMI 1640 medium (Invitrogen) and were labeled in serum-free medium with CM-Dil (1 g/ml) at 37 °C for 30 minutes, followed by at 4 °C for 10 minutes. After shearing, ECs were incubated with the labeled THP-1 cells at a concentration of 5 × 10^5^ THP-1 cells per milliliter (in a total of 4 ml) for 30 minutes at 37 °C. Non-adherent monocytes were washed away with 1640, and the adherent monocytes were fixed with 4% paraformaldehyde for 5 minutes. Cells were counted in 10 randomly selected microscopic fields under an inverted epi-fluorescence microscope.

### Cell proliferation measured by Ki67 staining

Standard fluorescent immunocytochemistry was employed to assay cell proliferation, as indicated by a positive staining of proliferative marker, Ki67. The numbers of stained cell nuclei were scored in 10 randomly selected microscopic fields and the ratios of stained to total cells (DAPI-positive cells) expressed as numerical value relative to the CL/PS were defined as Ki67 positive staining.

### Methylation specific-polymerase chain reaction (MSP)

Genomic DNA was extracted for methylation analysis from cells after exposure to PS or OS for 6 hours. One gram of genomic DNA was modified with sodium bisulfite using the EpiMark Bisulfite kit (BioLabs) according to the manufacturer’s instructions. Polymerase chain reaction (PCR) amplifications were carried out in a total volume of 25 μL by using 2× EasyTaq PCR SuperMix (TRANSGEN BIOTECH). The primer sets are listed in Table S1. MSP reactions were subjected to initial incubation at 95 °C for 5 minutes, followed by 40 cycles of 95 °C for 30 seconds, and annealing at the appropriate temperature for 30 seconds and 72 °C for 30 seconds. Final extension was done by incubation at 72 °C for 5 minutes. MSP products were separated on 2% agarose gels and visualized after Gel-Red (Beyotime) staining.

### Carotid partial ligation and vessel harvesting

All animal studies were performed in accordance with the approved protocol of the Animal Care and Use Committee of Peking University and were performed in accordance with the “Guide for the care and use of laboratory animals” published by the US National Institutes of Health (publication No. 85–23, revised 1996). The surgical procedures are modified from the previous reports^3,8^. Briefly, the left carotid bifurcation of mice was exposed following a neck incision. Three branches (external carotid, internal carotid, and occipital) of the left carotid artery were ligated with a 6-0 silk suture, and the superior thyroid artery was left intact. The right carotid arteries were served as control. At one week after operation, the animals were sacrificed and the vessels were perfused with a fixative (4% paraformaldehyde in PBS) under pressure (100 mmHg). The arteries were dissected out and further fixed by immersion in the fixative for 16 hours before being embedded in Tissue-Tek OCT compound and stored in −80 °C.

### *In vivo* delivery of virus

ApoE−/− and C57BL/6 wildtype mice (8–12 weeks old, 18–25 g) were anesthetized and were then subjected to partial ligation of carotid artery. After the surgery, some mice were injected intraperitoneally twice per week with rapamycin (1 mg/kg/day) or with control reagent. Some mice received a locally intraluminal incubation of ad-*m*shDNMT1 (Gene Pharma) or control virus. Briefly, left common carotid artery was exposed by blunt dissection. The internal carotid and occipital artery were ligated with 6–0 silk suture. Heart proximal end of the left common carotid artery was clamped temporarily. Adenovirus was injected into the common carotid artery through external carotid artery with a catheter and was retained intraluminally in the artery for 30 minutes. After that the external carotid artery was ligated while the superior thyroid artery was left intact. The clamp was removed. ApoE−/− mice were fed with a Western diet (D12108C high fat rodent diet with 1.25% cholesterol) immediately after surgery. One or four weeks after ligation, the mice were sacrificed and fixed for 5 minutes by perfusion through left cardiac ventricle with 4% paraformaldehyde in PBS under physiological pressure. The ligated carotid arteries were harvested and subjected to histology and immunostaining analyses of the vessels.

### Migration assay

EC migration was evaluated with scratch wound healing assay. In brief, the cells were subjected to either PS or OS for 6 hours. Then the monolayer was scratched using a pipette tip, and the cells were then allowed to migrate for 12 hours. Wound closure rate of the scratch was measured to quantitatively evaluate cell migration. (Wound closure rate =  (A_0_ − A _t_) /A_0_ × 100%, A_0_: mean width of scratches at 0 hour, A_t_: mean width of scratches at 12 hours).

### ROS production assay

ROS production was detected with DHE kit (KeyGEN BioTECH) according to the manufacturer’s instructions. In brief, ECs were subjected to either PS or OS for 6 hours, and then the cells were incubated in DHE (25 μM in M199 basal medium) for 30 minutes at room temperature. The cells were then visualized by epi–fluorescence microscopy.

### Chromatin immunoprecipitation

ECs were subjected to either PS or OS for 6 hours. After shearing, the cells were washed once with PBS (room temperature), fixed with 1% formaldehyde in PBS for 10 minutes, rinsed twice with ice-cold PBS, and scraped into 1 ml of ice-cold PBS. The pellet was resuspended with 300 μl of lysis buffer (1% SDS, 5 mM EDTA, 50 mM Tris-HCl [pH 8.1], and protease inhibitors), incubated on ice for 10 minutes, and sonicated for 3 times at 12 seconds each. A 10% aliquot was saved as an input. The lysate was 1:10 diluted in dilution buffer (1% Triton X-100, 2 mM EDTA, 150 mM NaCl, 20 mM Tris-HCl [pH 8.1], and protease inhibitors), and was incubated with antibody against DNMT1 (Santa Cruz Biotech) for 6 hours or overnight at 4 °C and then with 30 μl of protein A-G sepharose beads for another 2 hours. The sepharose beads were washed sequentially with buffer TSE I (0.1% SDS, 1% Triton X-100, 2 mM EDTA, 20 mM Tris.HCl, pH 8.1, 150 mM NaCl), buffer TSE II (0.1% SDS, 1% Triton X-100, 2 mM EDTA, 20 mM Tris.HCl, pH 8.1, 500 mM NaCl), buffer III (0.25 M LiCl, 1% NP-40, 1% deoxycholate, 1 mM EDTA, 10 mM Tris.HCl, pH 8.1), and TE buffer. The beads were then treated with RNase A (50 μg/mL) and proteinase K (7.5 μL of 20 mg/mL) at 37 °C for 30 minutes. Cross-links were reversed at 65 °C overnight. DNA was extracted with DNA Pure-Spin Kit(Vigorous), and subjected for PCR amplification. The primer sets are listed in Table S1.

### siRNA-mediated gene silencing

ECs were transfected with lipofectamine 2000 reagent according to the manufacturer’s instructions. The sequences of siRNAs are listed in Table S1.

### Statistical Analysis

Data are expressed as mean ± SEM from at least 3 independent experiments. Statistical analysis was performed by unpaired or paired t-test for 2 groups of data and by 1-way ANOVA for multiple comparisons. Statistical significance among multiple groups was determined by post hoc analysis (Tukey honestly significant difference test). Values of P < 0.05 were considered statistically significant.

### Data Availability Statement

The datasets generated during and/or analysed during the current study are available from the corresponding author on reasonable request.

## Electronic supplementary material

Supplementary Information
