# Supplementary material for: The Mammalian Target of Rapamycin and DNA methyltransferase 1 axis mediates vascular endothelial dysfunction in response to disturbed flow
Source: Sci Rep. 2017 Nov 8;7:14996. doi: 10.1038/s41598-017-15387-5 (PMC5678172; doi:10.1038/s41598-017-15387-5)
Supplement: Supplementary file 1 — Supplementary Information [file 41598_2017_15387_MOESM1_ESM.pdf]

Supplementary Information

Title: The Mammalian Target of Rapamycin and DNA methyltransferase 1 axis mediates vascular endothelial dysfunction in response to disturbed flow

Yun-Peng Zhang#, Yi-Tao Huang#,Tse-Shun Huang, Wei Pang, Juan-Juan Zhu, Yue-Feng Liu, Run-Ze Tang, Chuan-Rong Zhao, Wei-Juan Yao, Yi-Shuan Li, Shu Chien, and Jing Zhou*

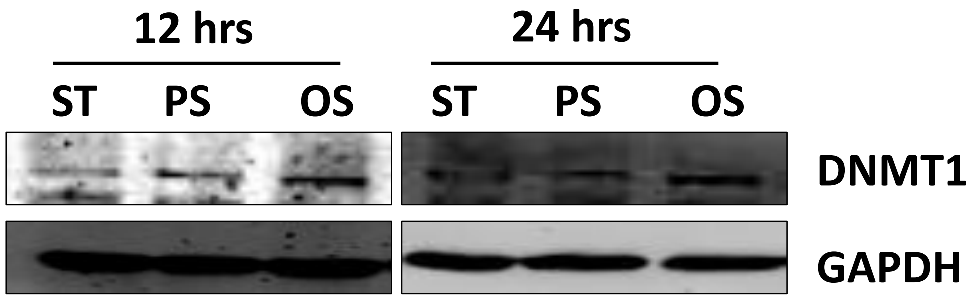

Figure S1

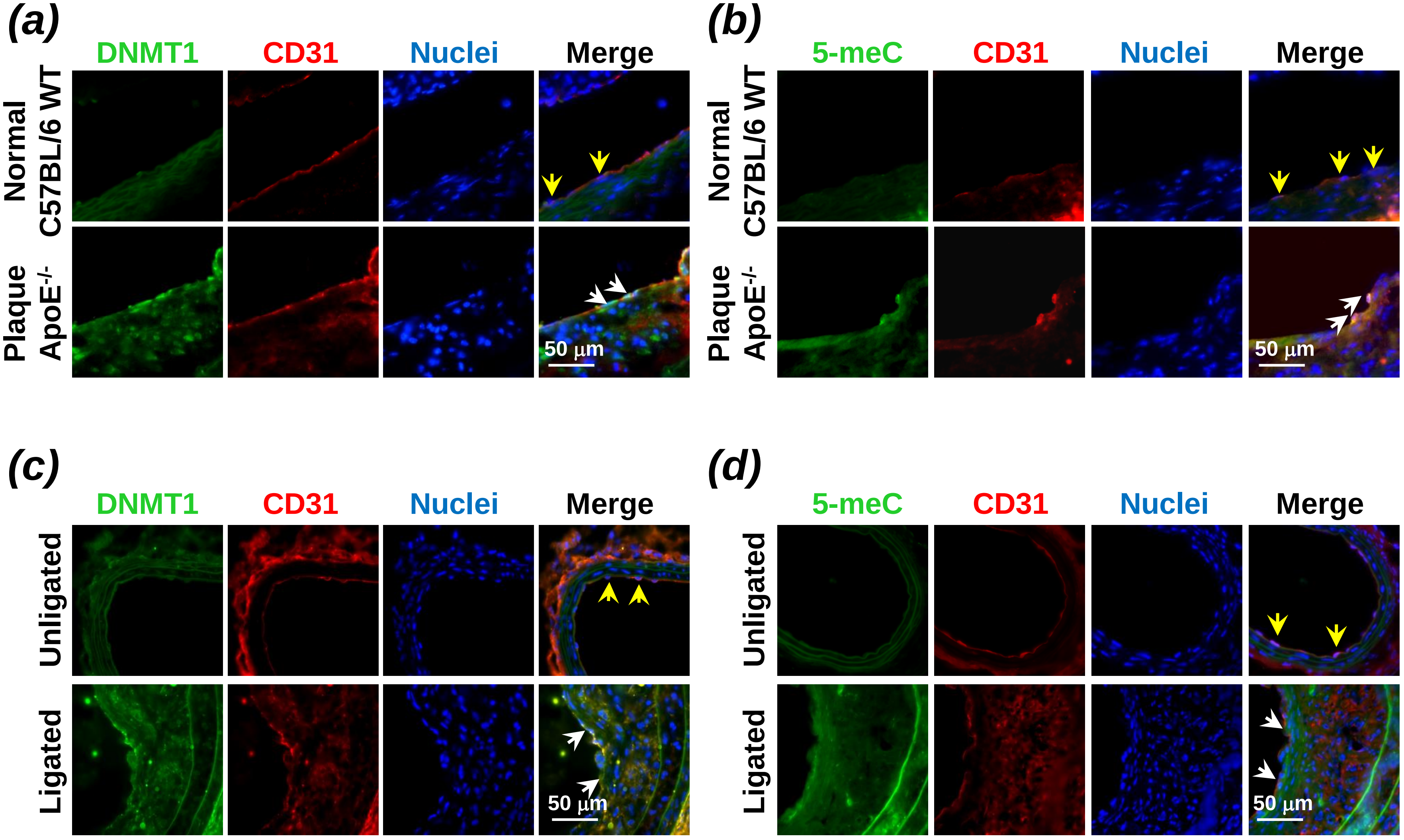
(a)
DNMT1
CD31
Nuclei
Merge
Normal C57BL/6 WT
Plaque ApoE-/-
50 μm
(b)
5-meC
CD31
Nuclei
Merge
Normal C57BL/6 WT
Plaque ApoE-/-
50 μm
(c)
DNMT1
CD31
Nuclei
Merge
Unligated
Ligated
50 μm
(d)
5-meC
CD31
Nuclei
Merge
Unligated
Ligated
50 μm

Figure S2

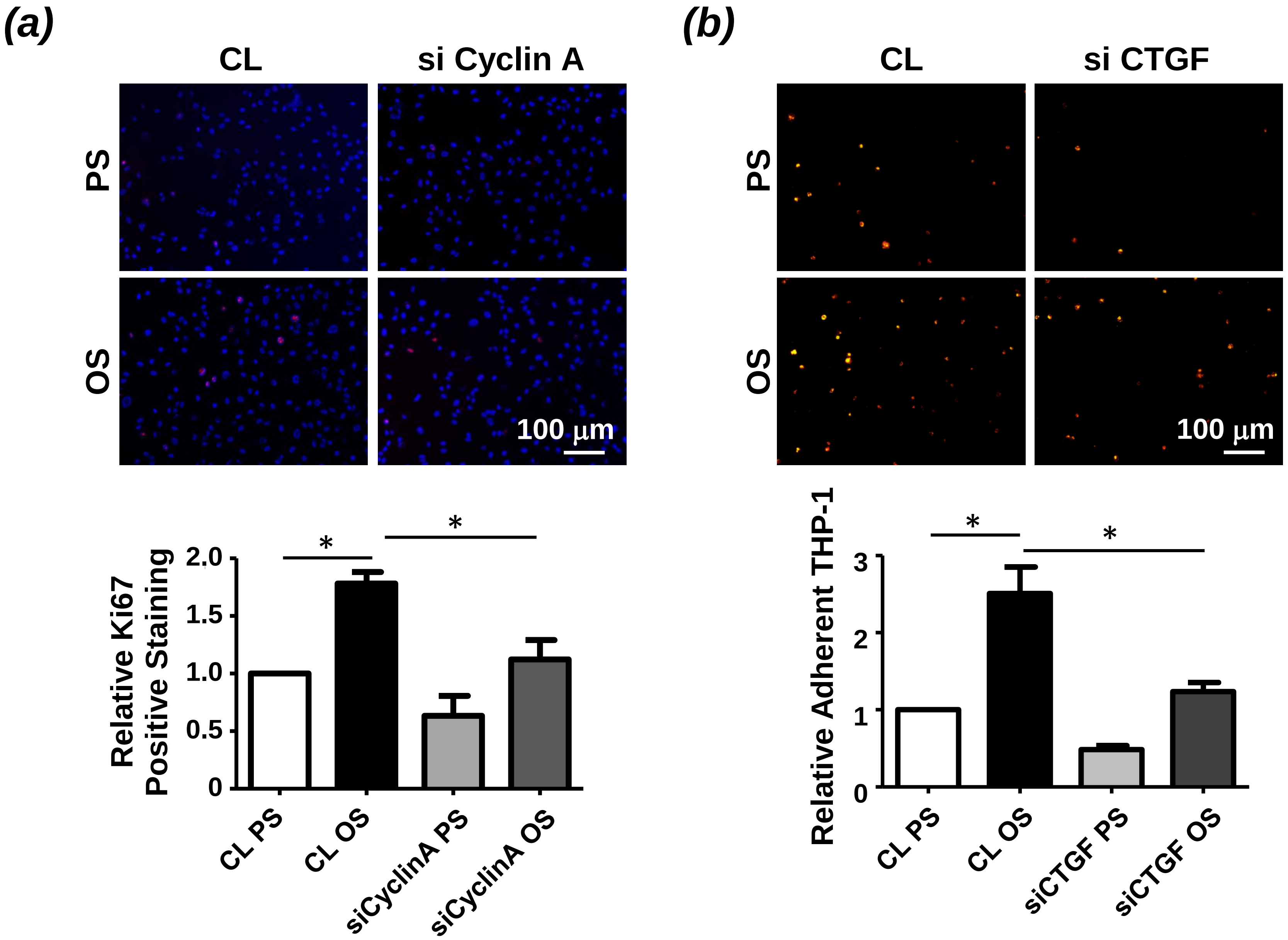

(a)
CL
si Cyclin A
PS
OS
100 μm
*
*
2.0
1.5
1.0
0.5
0
Relative Ki67 Positive Staining
CL PS
CL OS
siCyclinA PS
siCyclinA OS
(b)
CL
si CTGF
PS
OS
100 μm
*
*
3
2
1
0
Relative Adherent THP-1
CL PS
CL OS
siCTGF PS
siCTGF OS

Figure S3

Primers and siRNAs

Name	Sequences
Cyclin A-f (ChIP)	CGGACAGCCTCGCTCACTA
Cyclin A-r (ChIP)	AGCCAAAG ACGCCCAGAG
CTGF-f (ChIP)	TGGTGCTGGAAATACTGCGC
CTGF-r (ChIP)	ACATTCCTCGCATTCCTCCC
CTGF-m-f (MSP)	AGTGTTAAGGGGTTAGGATTAATTC
CTGF-m-r (MSP)	GCACTAACTATCTCCTCTCAACGA
CTGF-u-f (MSP)	GAGTGTTAAGGGGTTAGGATTAATTT
CTGF-u-r (MSP)	ACACTAACTATCTCCTCTCAACAAA
Cyclin A-m-f (MSP)	AAGGTTGGGTAATTTAAATGATAGTC
Cyclin A-m-r (MSP)	AAAAAAACAAACTAACTAAAACGAA
Cyclin A-u-f (MSP)	TTGGGTAATTTAAATGATAGTTGT
Cyclin A-u-r (MSP)	AAAAAAAACAAACTAACTAAAACAAA
si-FAK	CCGGUCGAAUGAUAAGGUGUA
si-ERK1	CCAAAGCUCUGGACUUAUU
si-ERK2	CCAAAGCUCUGGACUUAUU
si-MTOR	GCUCGUAAGUUGGGAUAACA
si-Cyclin A	GAGGACCAGGAGAAUAUCA
si-CTGF	GCACCAGCAUGAAGACAUACC
si-ITGB3	GCUCAUCUGGAAACUCCUCAUCACC

Table S1

Supplementary figure legends

Figure S1, OS but nor PS induces DNMT1 expression. ECs were exposed to pulsatile shear (PS, 12 ± 4 dynes/cm^2) or OS (0.5 ± 4 dynes/cm^2) for indicated time, and expression of DNMT1 was analyzed by Western blot.

Figure S2, DNMT1 expression and global DNA methylation are elevated in the atherosclerotic endothelium in mice model. Single color images of Figure 2.

Figure S3, siRNA-mediated silencing of cyclin A or CTGF suppresses the OS-induced cell proliferation and monocyte adhesion. ECs were transfected with the indicated siRNAs and then exposed to pulsatile shear (PS, 12 ± 4 dynes/cm^2) or OS (0.5 ± 4 dynes/cm^2) for 6 hours, cell proliferation was indicated by immunofluorescent staining of the proliferative marker, Ki67 (a), and monocyte adhesion assay was performed to exam the cellular inflammatory response (b).

Uncropped images of Western blot

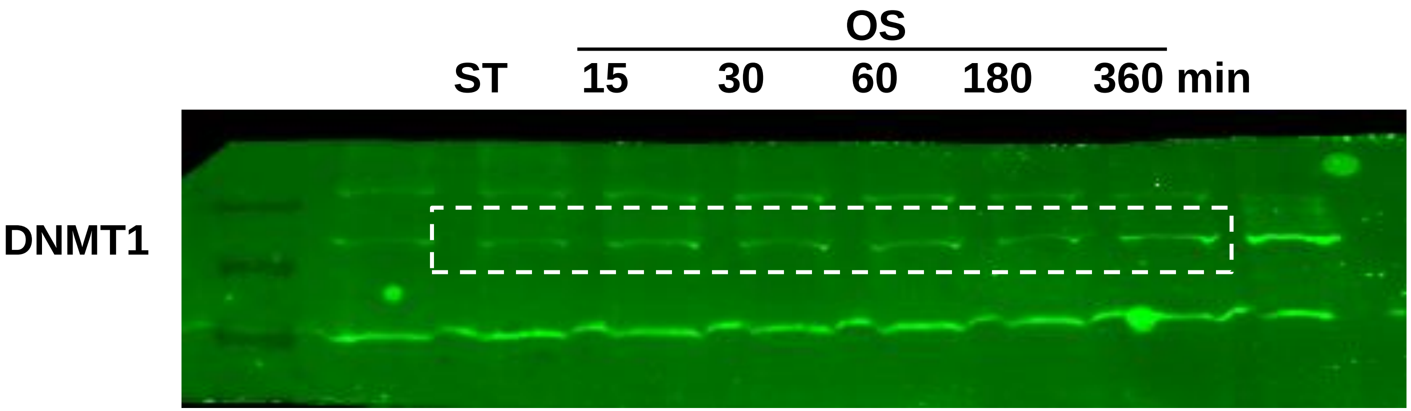

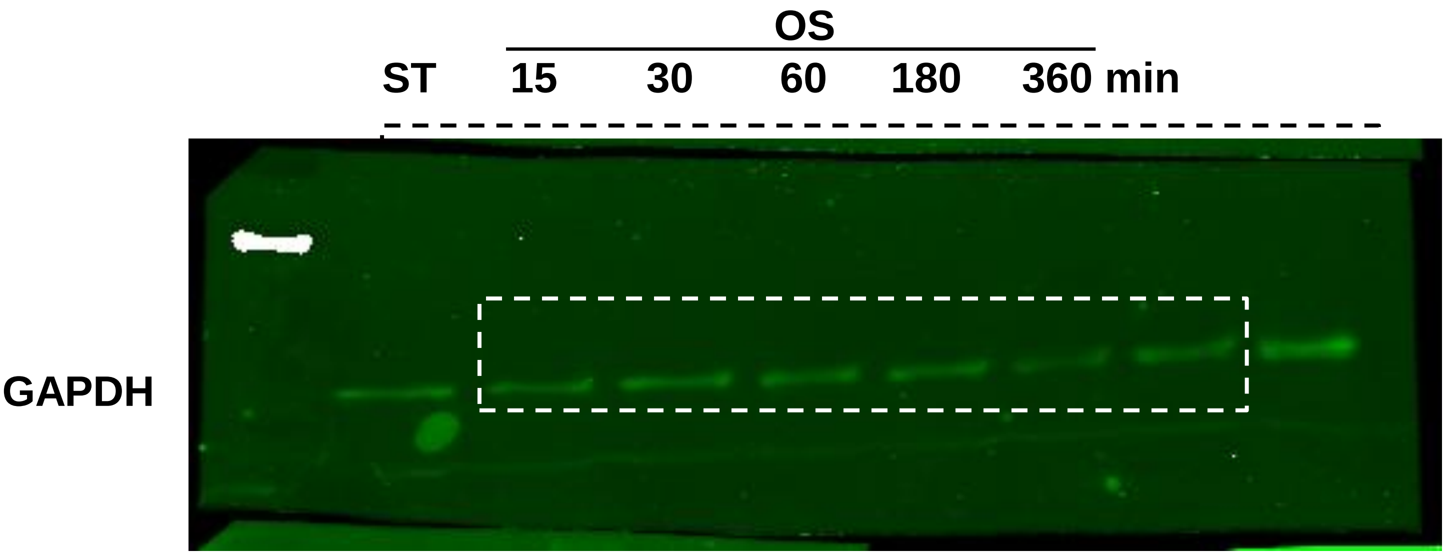

Figure1a

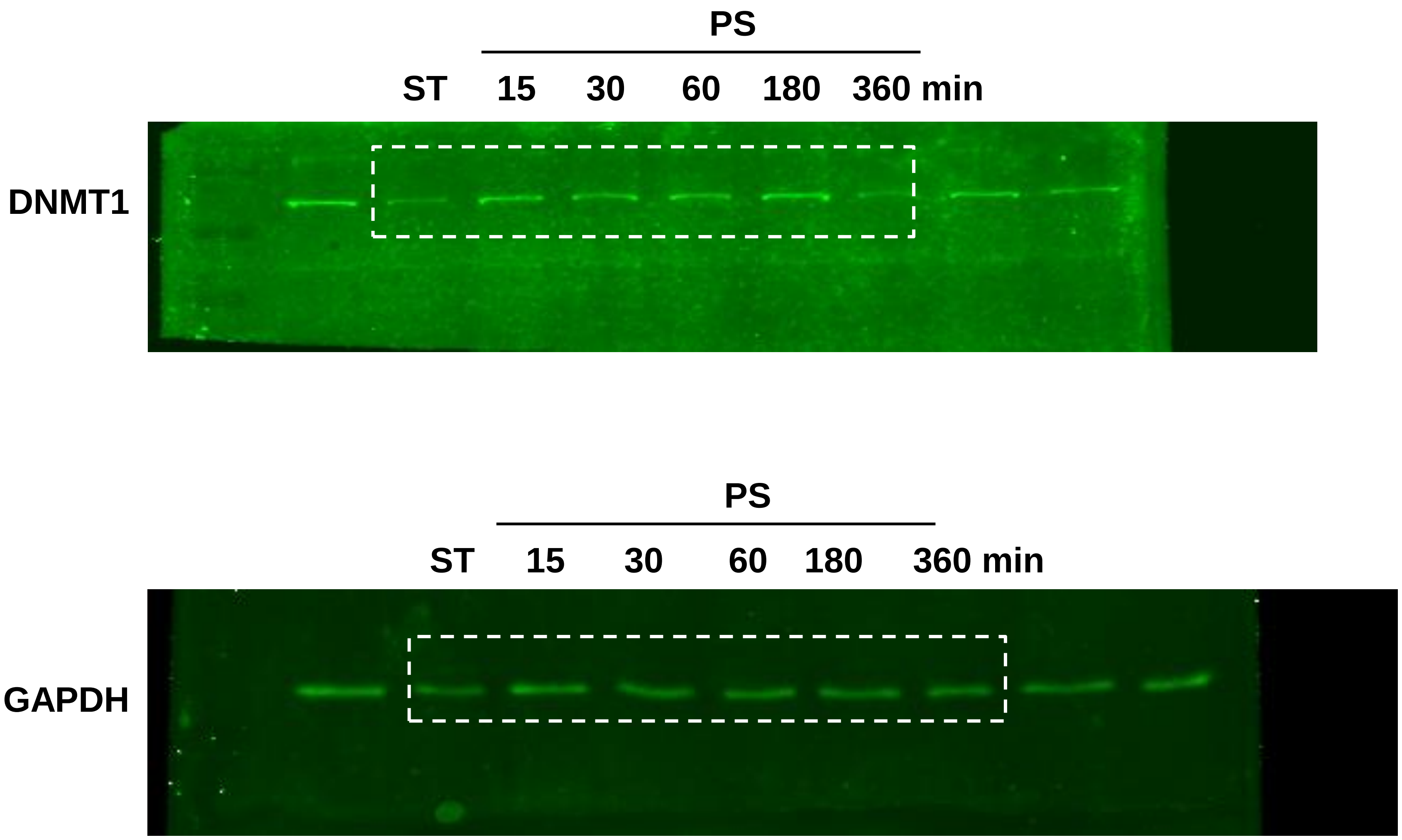

Figure1a

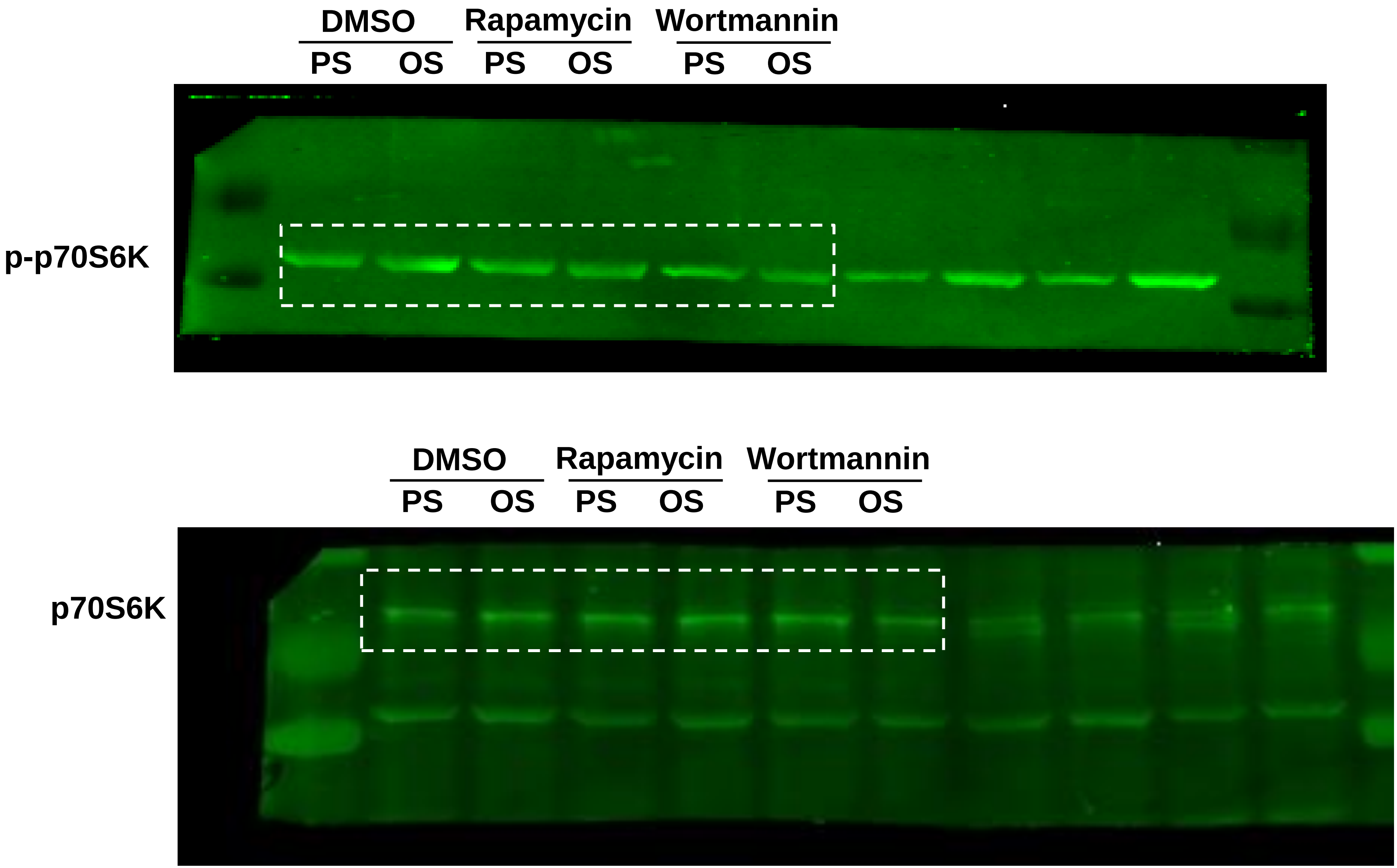
DMSO
Rapamycin
Wortmannin
PS
OS
PS
OS
PS
OS
p-p70S6K
DMSO
Rapamycin
Wortmannin
PS
OS
PS
OS
PS
OS
p70S6K

Figure4a

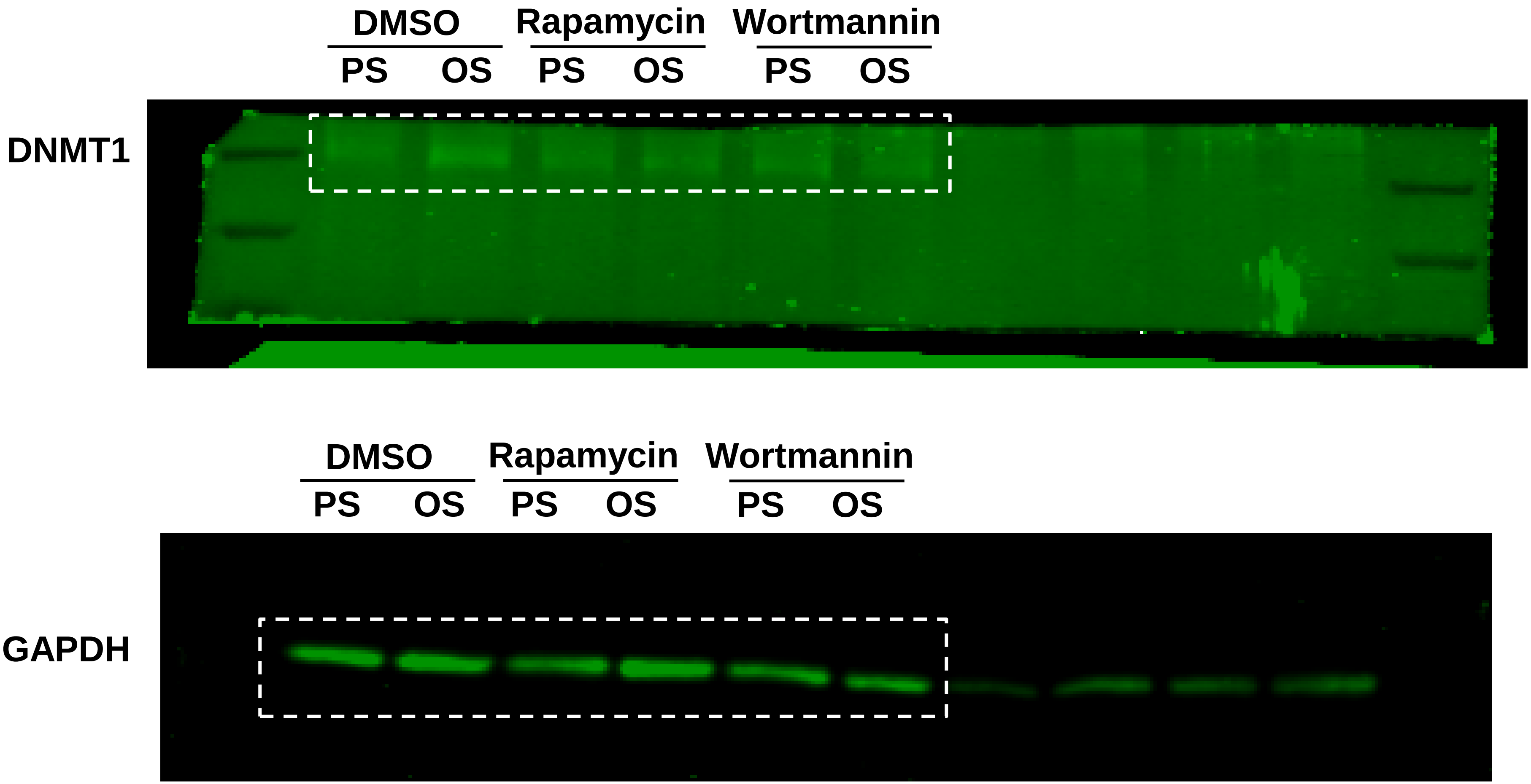
DMSO
Rapamycin
Wortmannin
PS
OS
PS
OS
PS
OS
DNMT1
DMSO
Rapamycin
Wortmannin
PS
OS
PS
OS
PS
OS
GAPDH

Figure4a

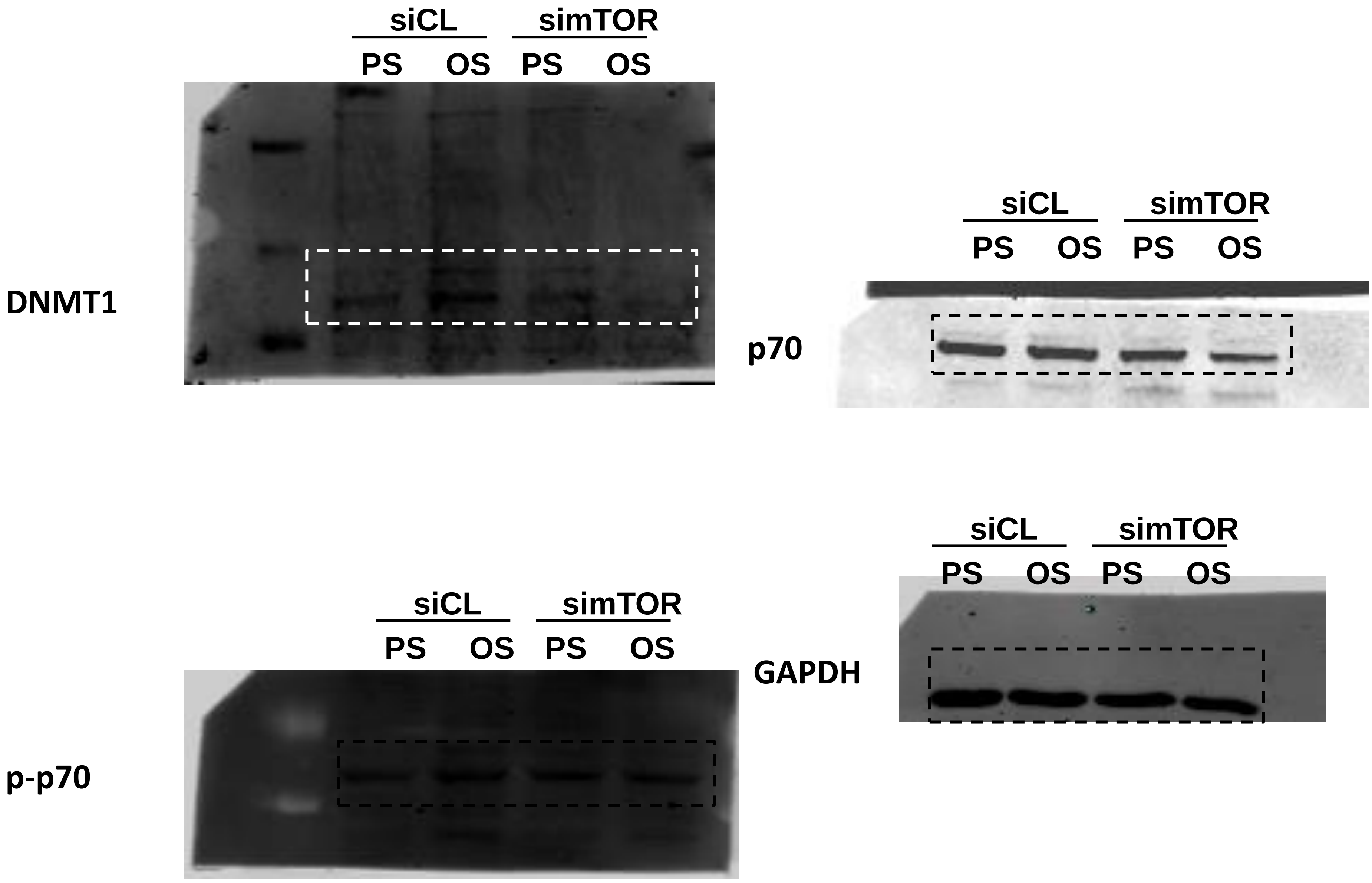
siCL
simTOR
PS
OS
PS
OS
DNMT1
siCL
simTOR
PS
OS
PS
OS
p70
siCL
simTOR
PS
OS
PS
OS
GAPDH
siCL
simTOR
PS
OS
PS
OS
p-p70

Figure4a

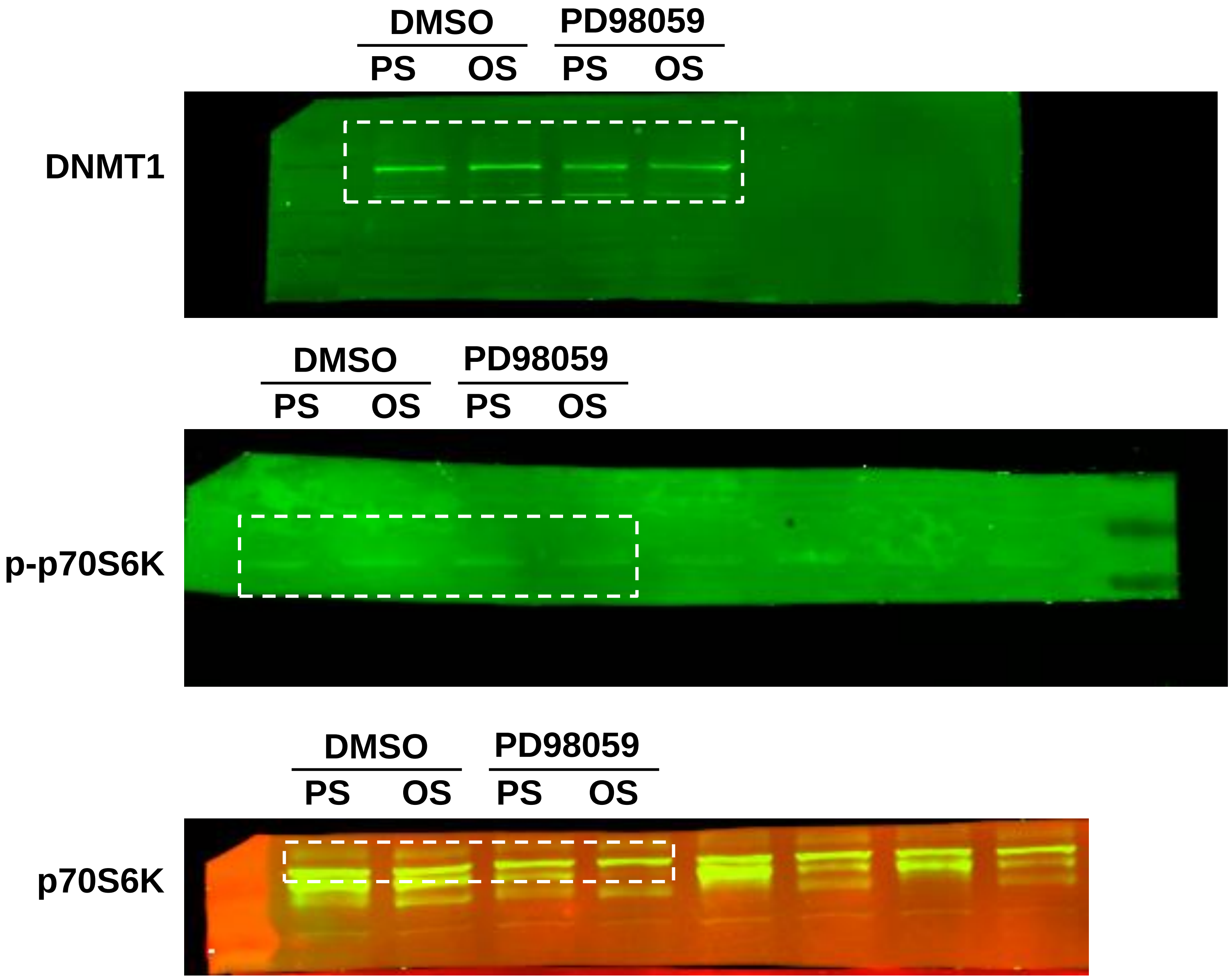
DMSO
PD98059
PS
OS
PS
OS
DNMT1
DMSO
PD98059
PS
OS
PS
OS
p-p70S6K
DMSO
PD98059
PS
OS
PS
OS
p70S6K

Figure4b

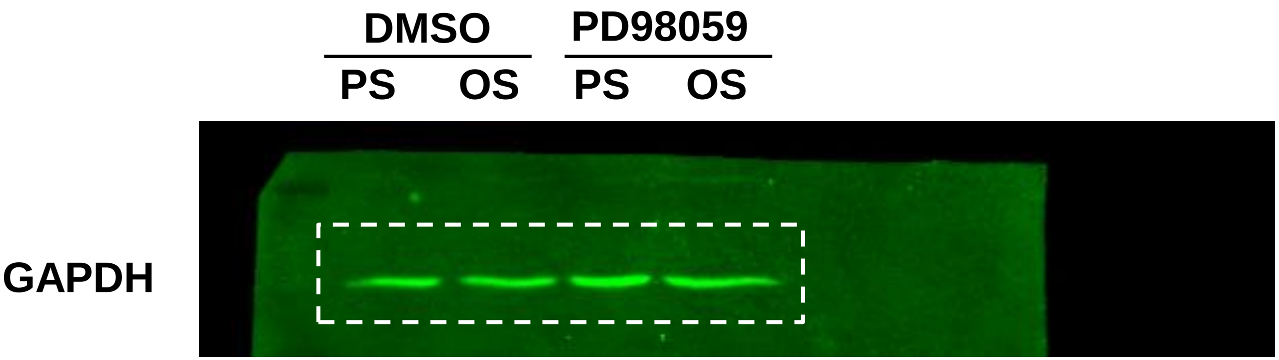

Figure4b

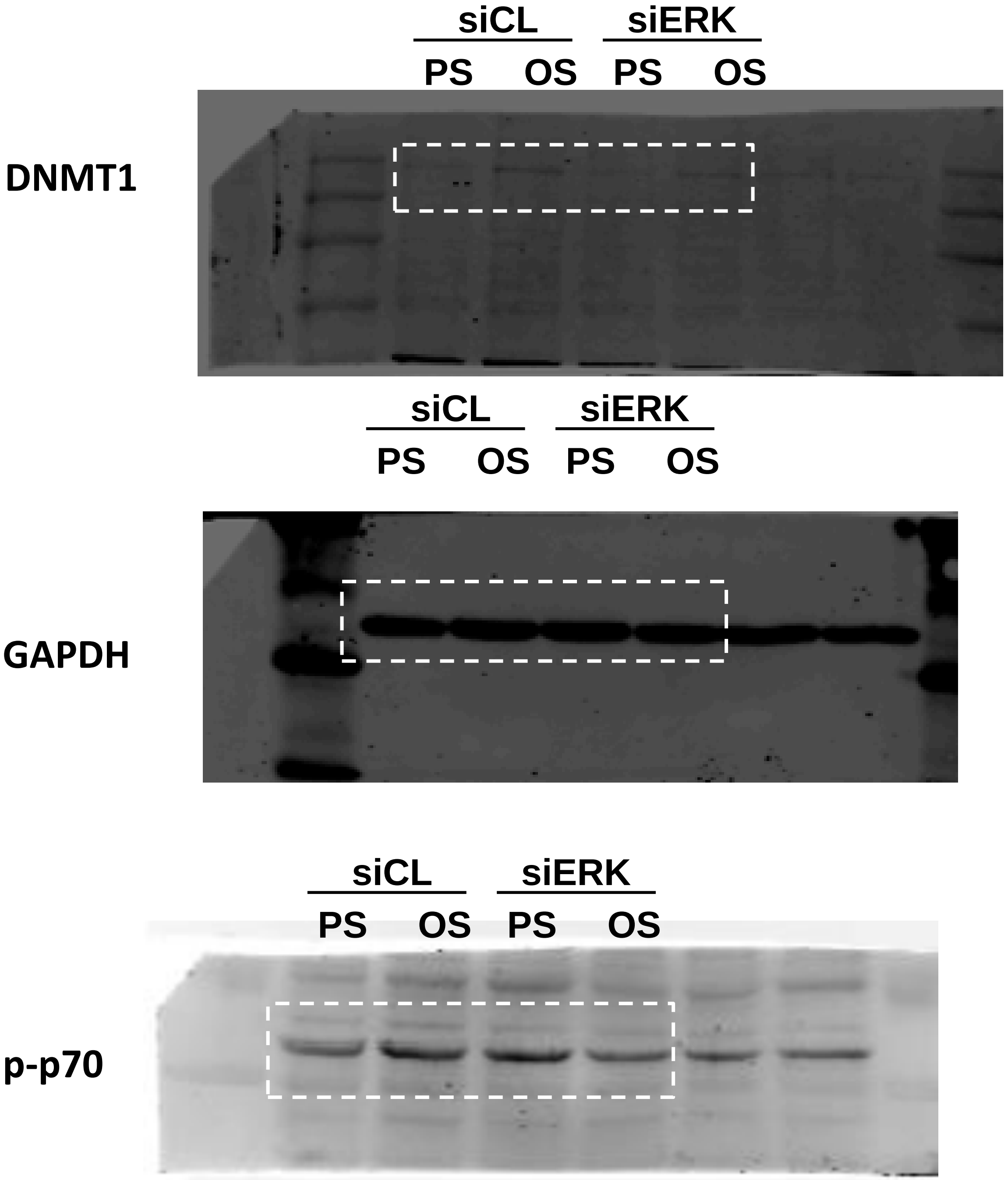
siCL
siERK
PS
OS
PS
OS
DNMT1
siCL
siERK
PS
OS
PS
OS
GAPDH
siCL
siERK
PS
OS
PS
OS
p-p70

Figure4b

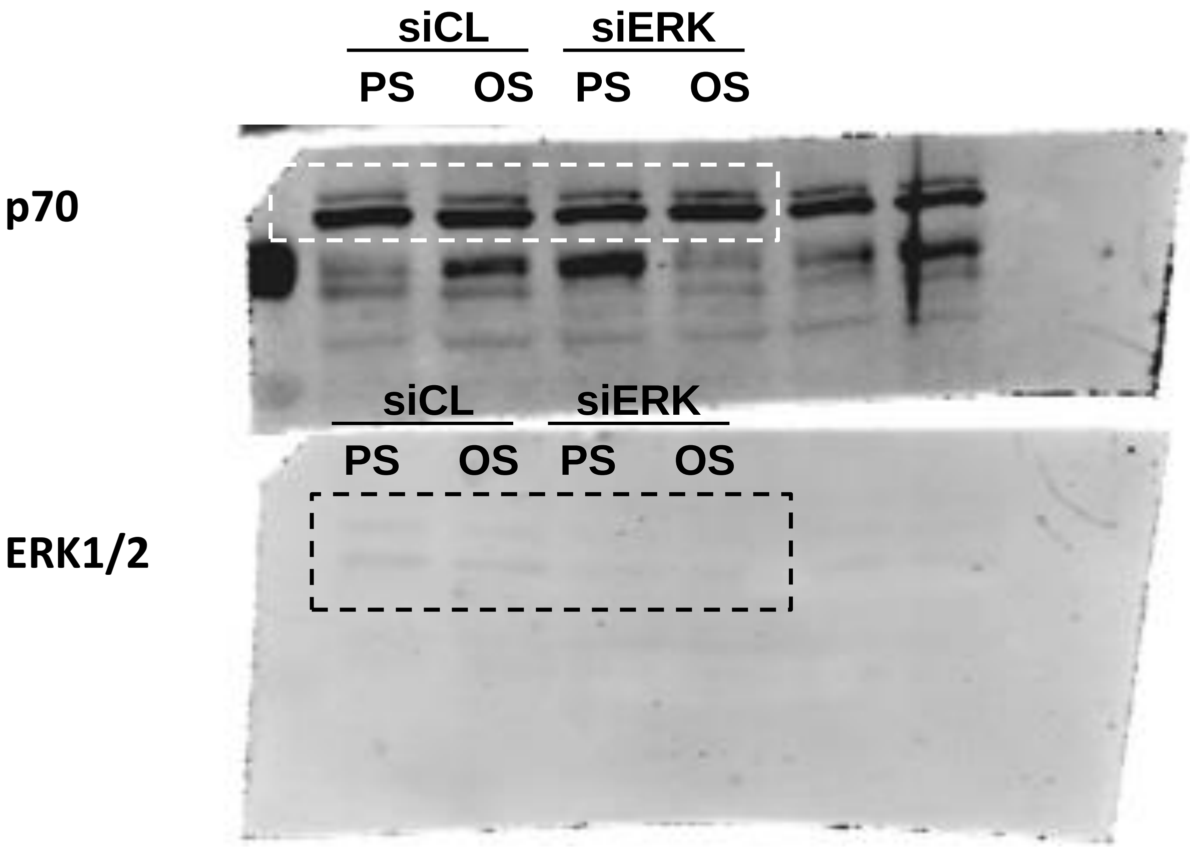

Figure4b

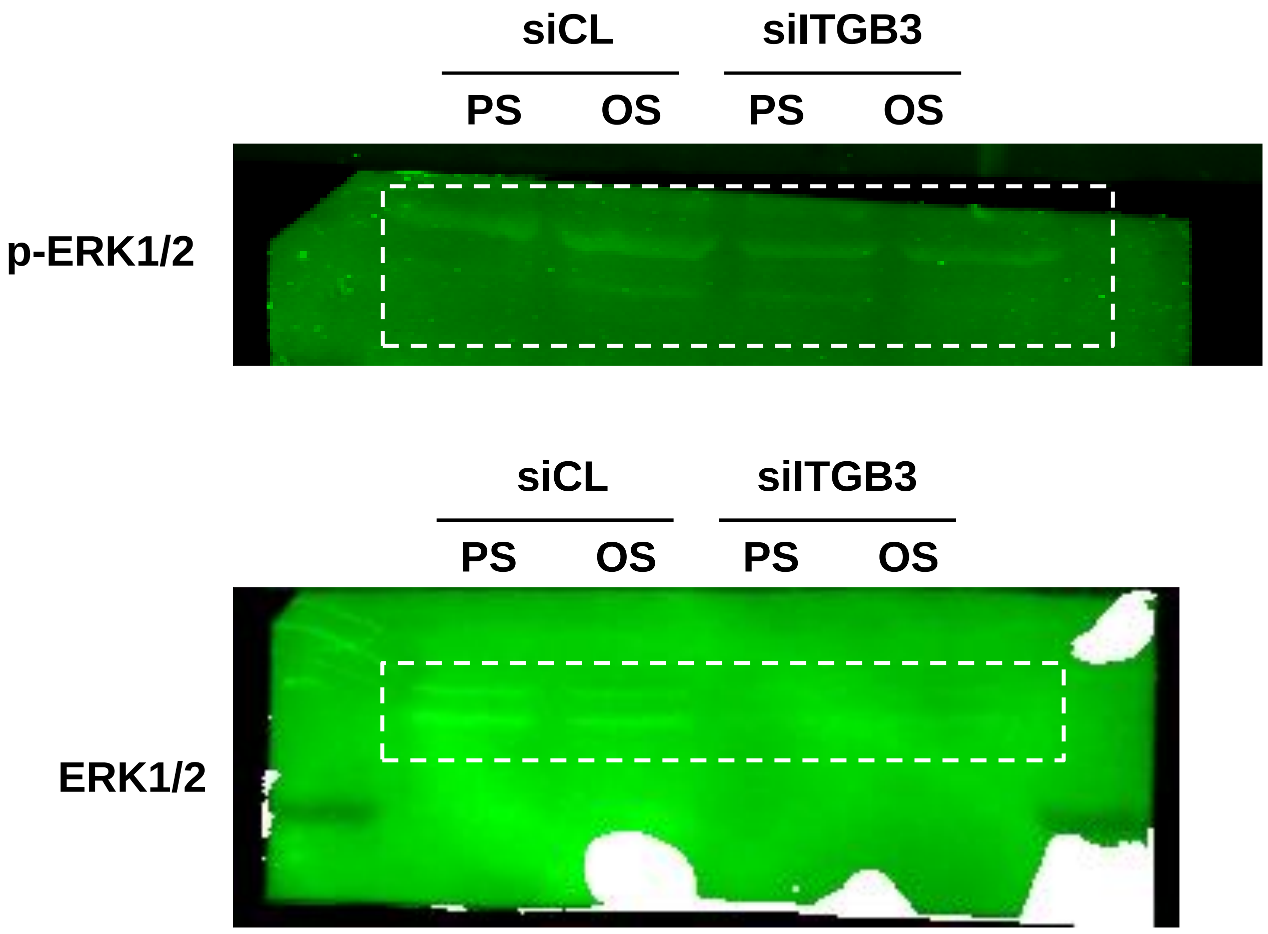

Figure4c

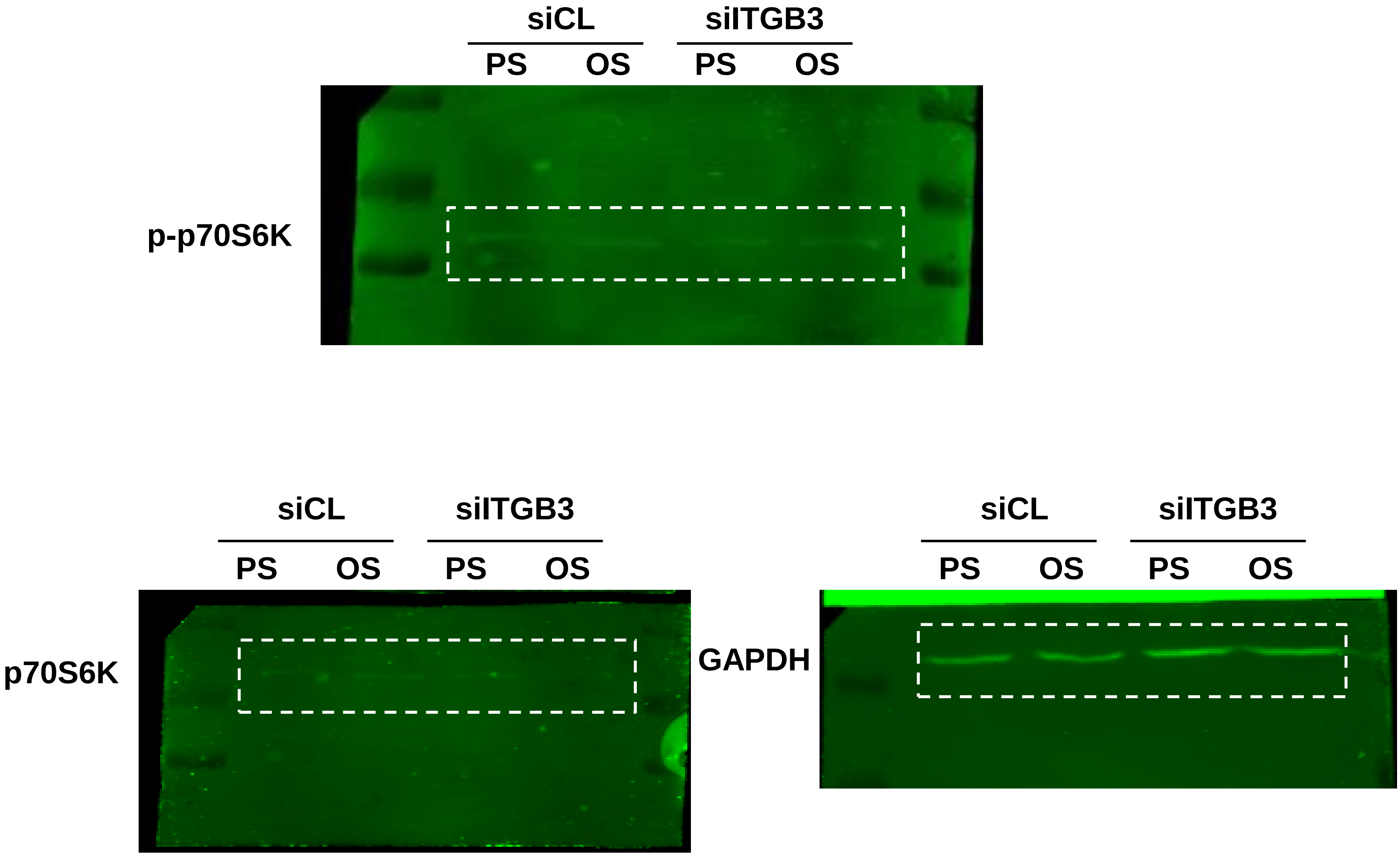
siCL
siITGB3
PS
OS
PS
OS
p-p70S6K
siCL
siITGB3
PS
OS
PS
OS
p70S6K
siCL
siITGB3
PS
OS
PS
OS
GAPDH

Figure4c

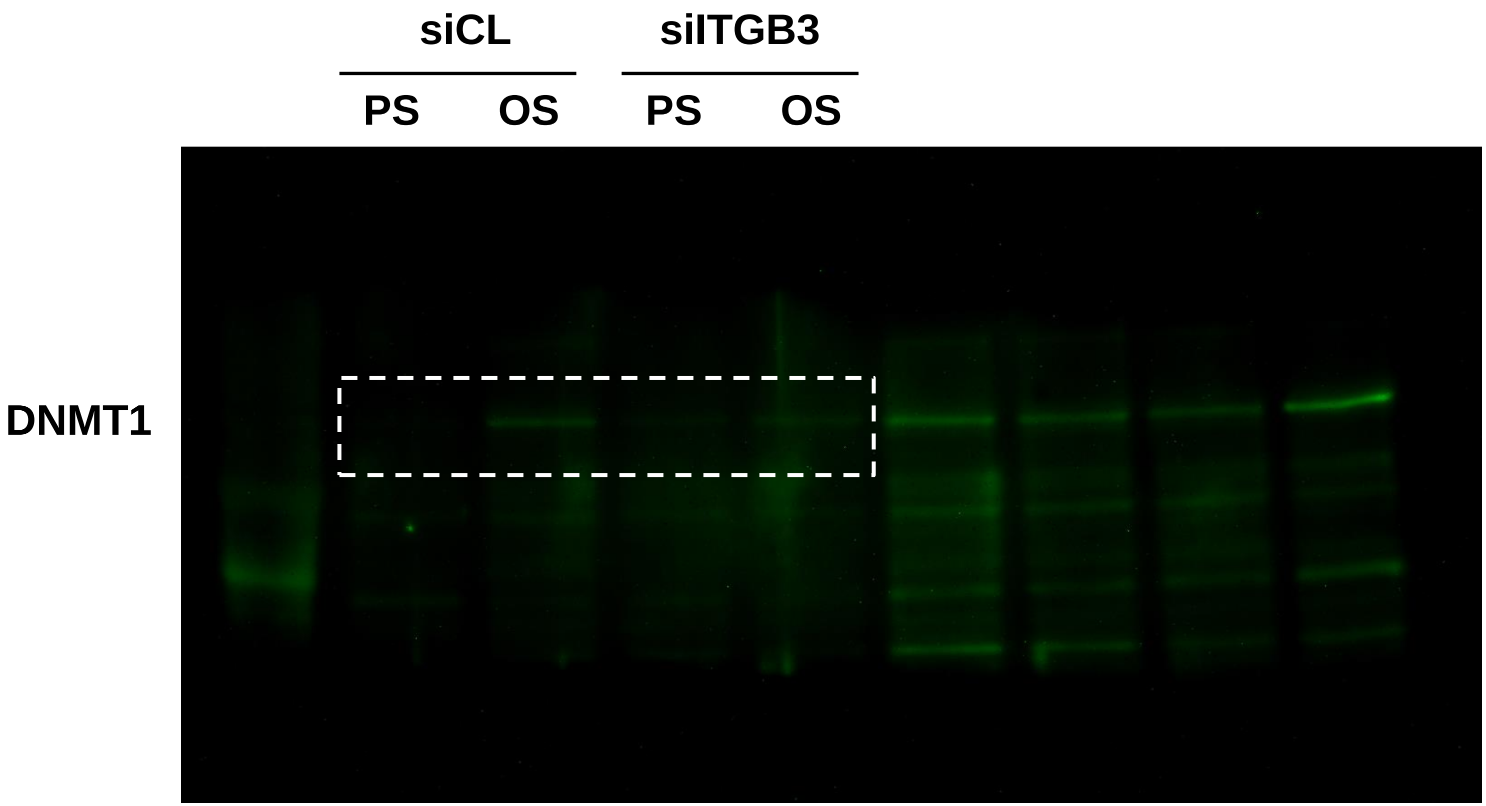

Figure4c

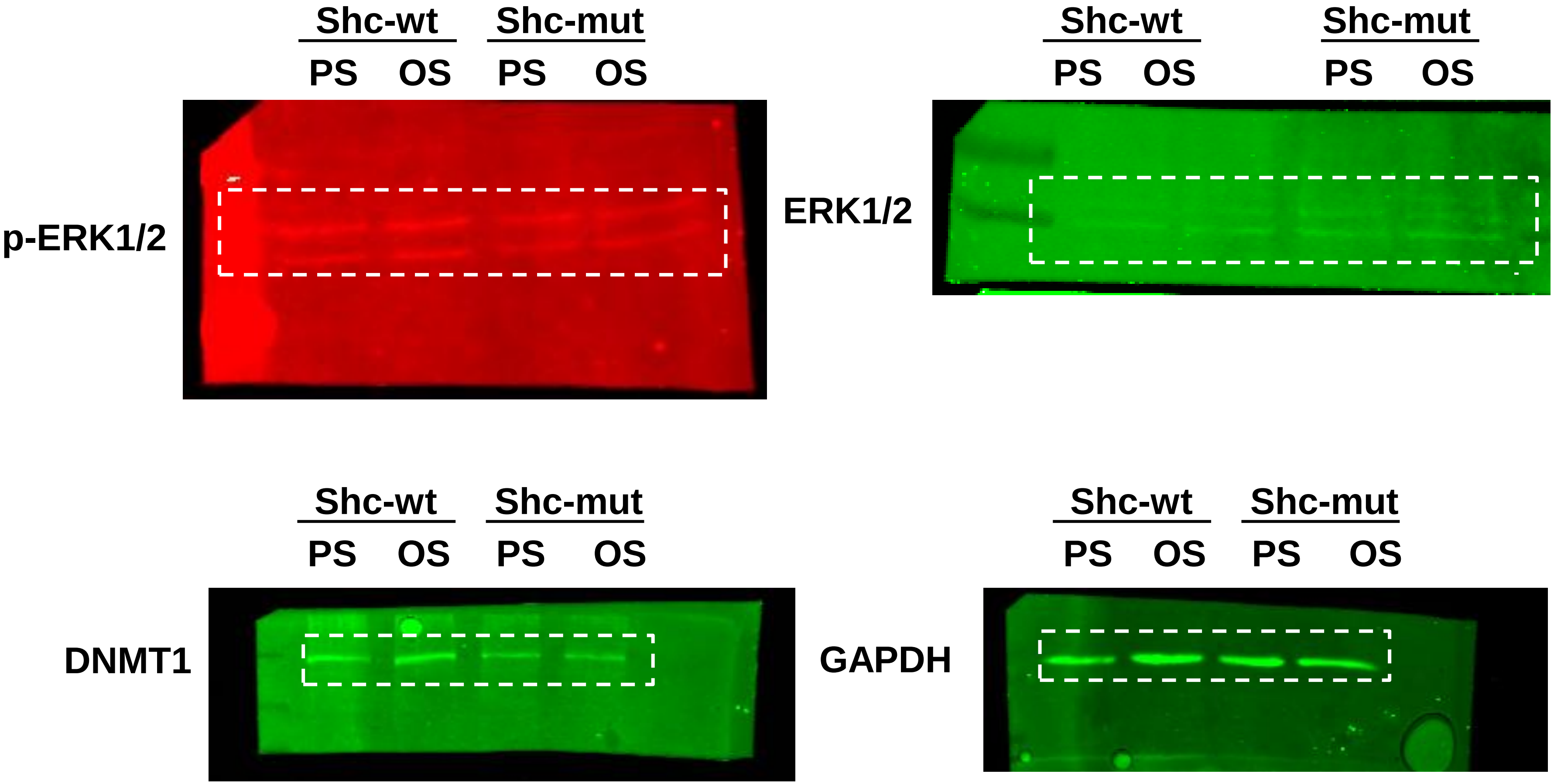
Shc-wt
Shc-mut
PS
OS
PS
OS
p-ERK1/2
Shc-wt
Shc-mut
PS
OS
PS
OS
ERK1/2
Shc-wt
Shc-mut
PS
OS
PS
OS
DNMT1
Shc-wt
Shc-mut
PS
OS
PS
OS
GAPDH

Figure4c

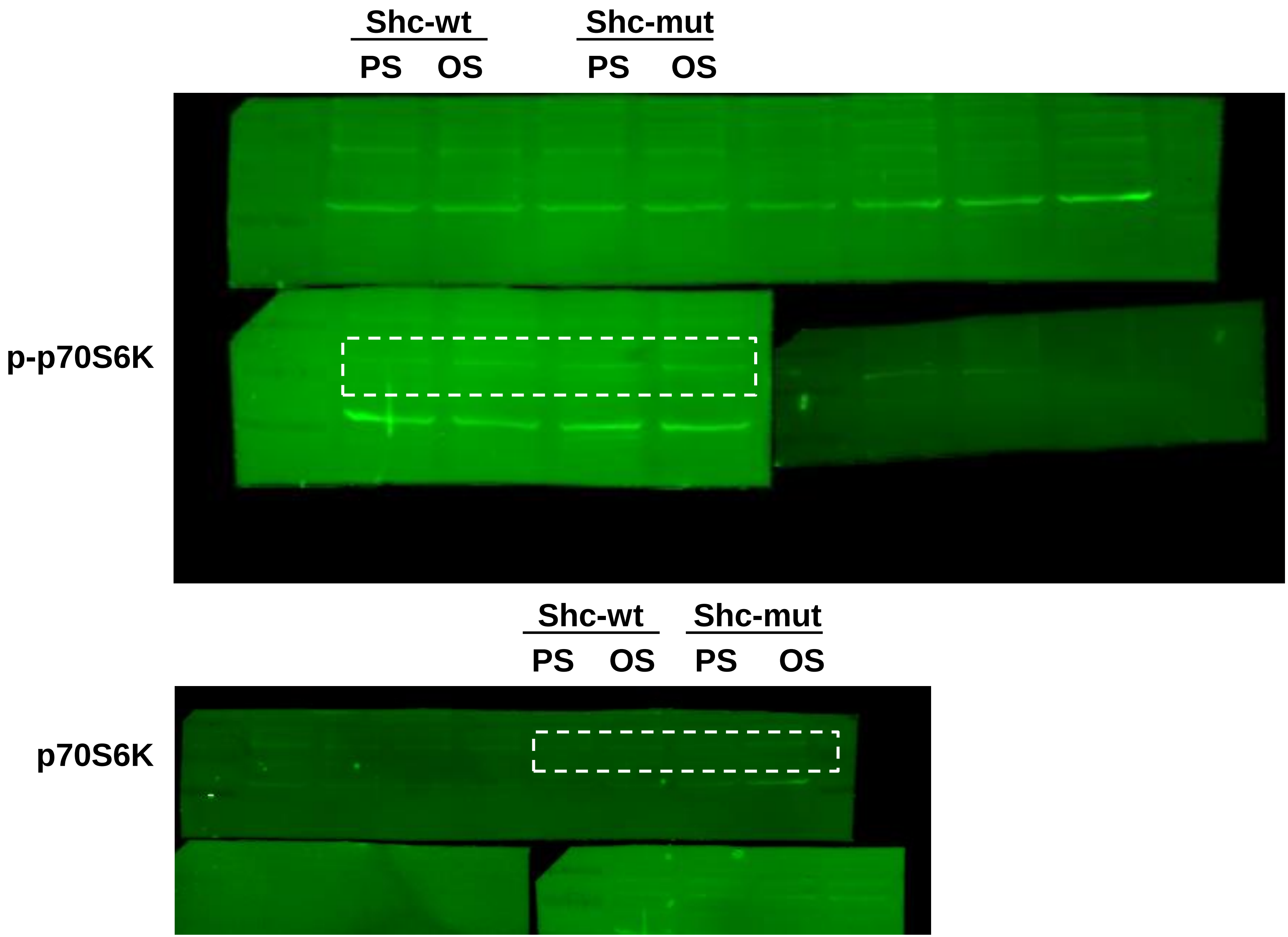
Shc-wt
Shc-mut
PS
OS
PS
OS
p-p70S6K
Shc-wt
Shc-mut
PS
OS
PS
OS
p70S6K

Figure4c

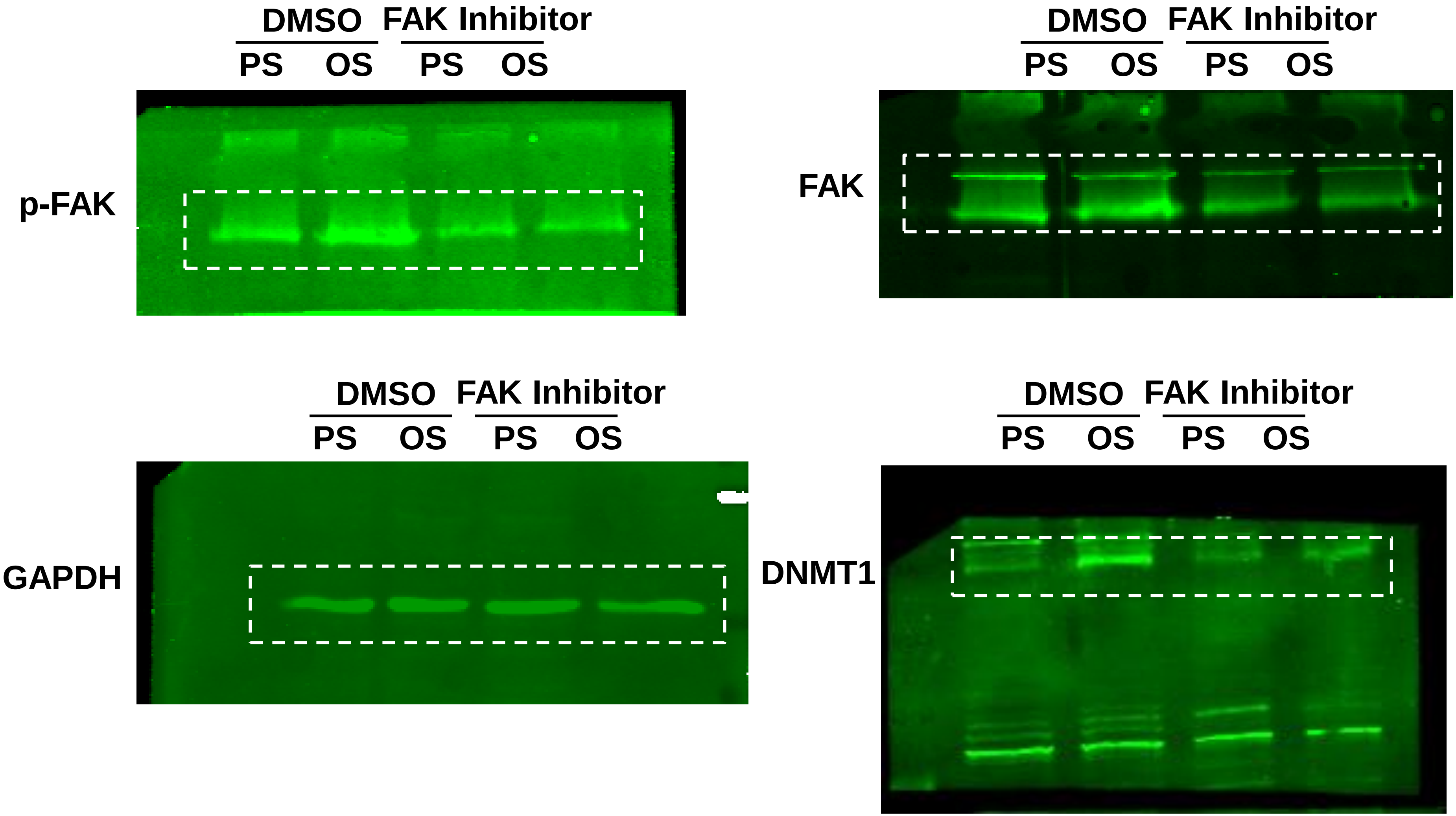

Figure4d

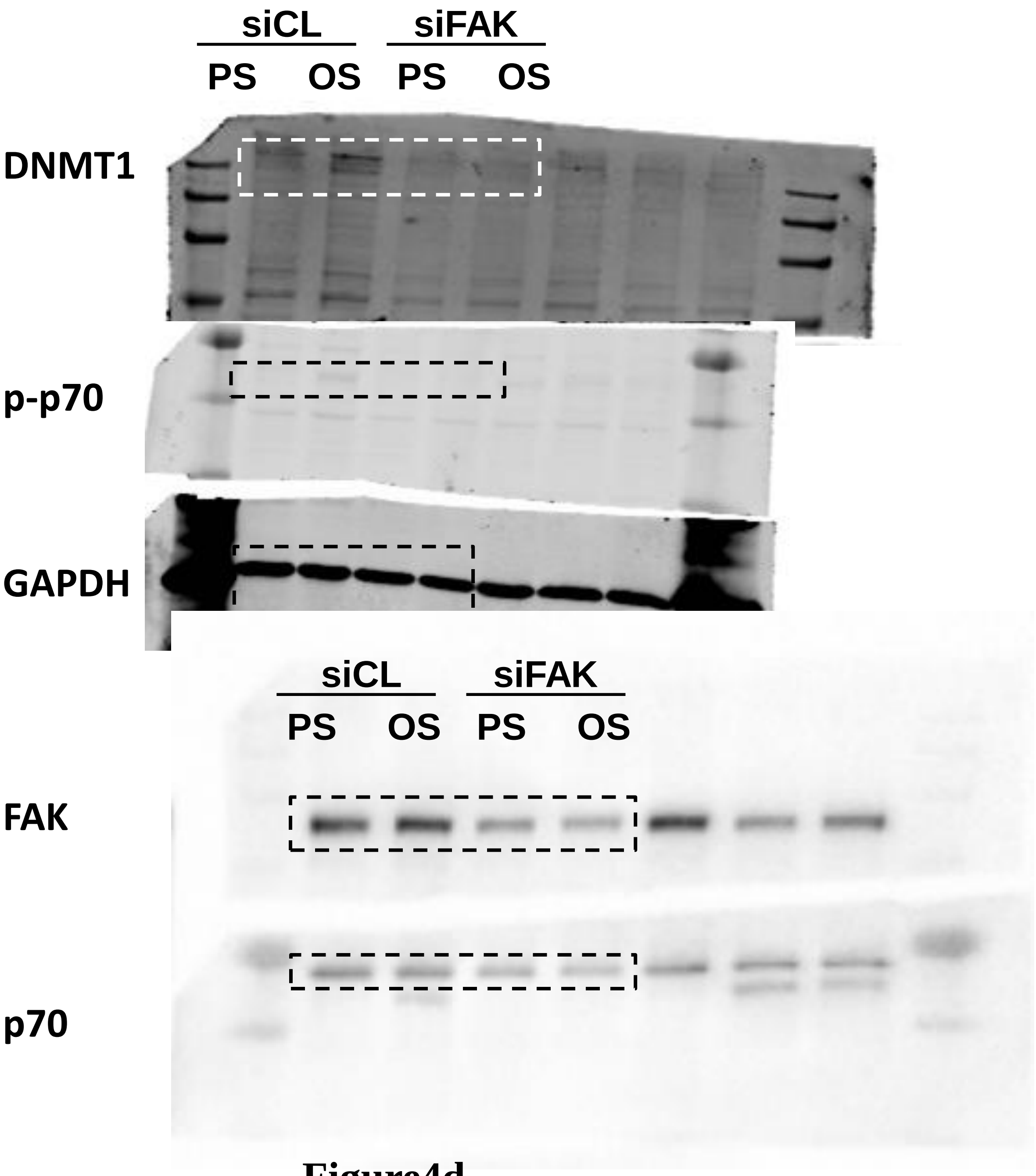
siCL
siFAK
PS
OS
PS
OS
DNMT1
p-p70
GAPDH
siCL
siFAK
PS
OS
PS
OS
FAK
p70

Figure4d

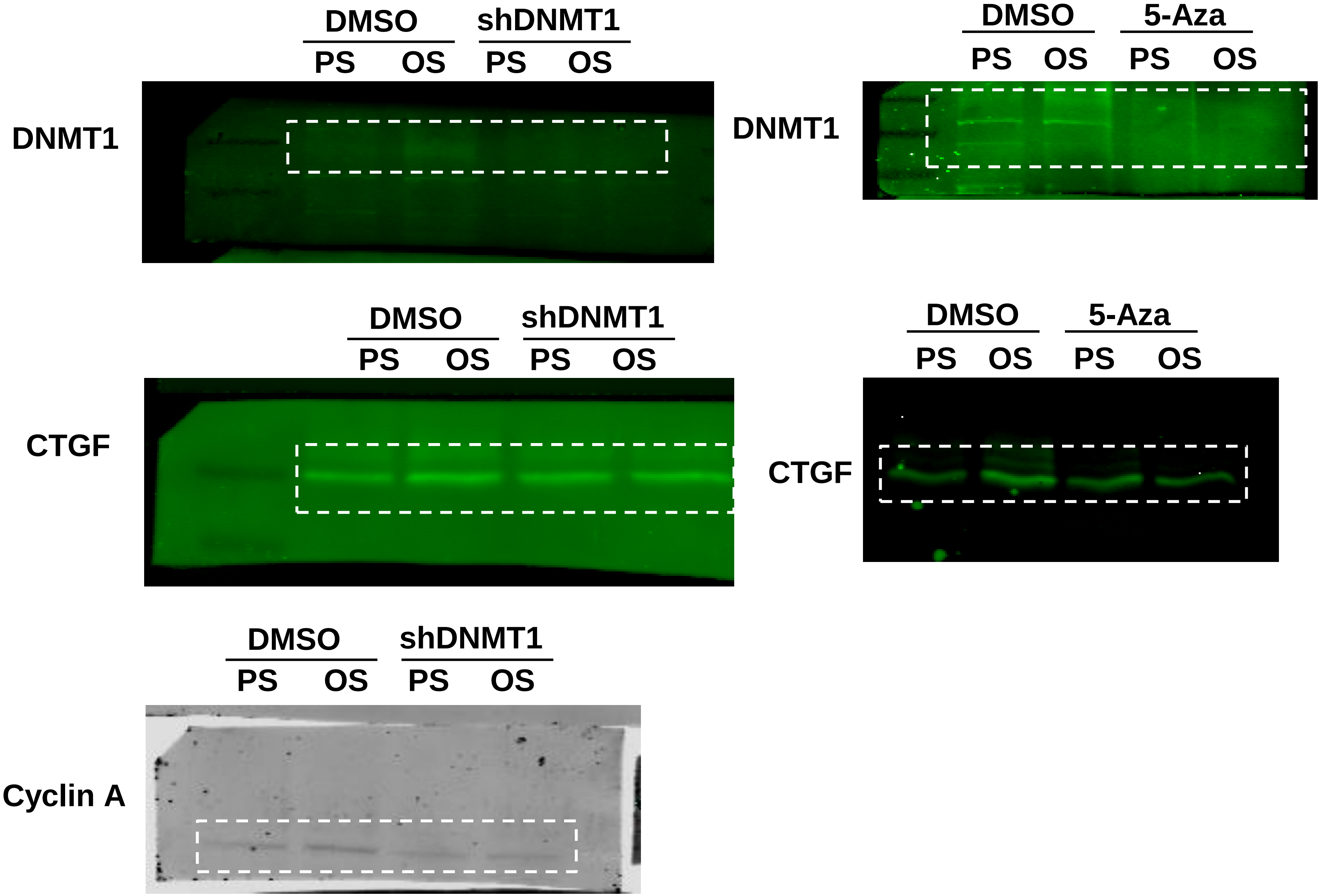
DMSO
shDNMT1
PS
OS
PS
OS
DNMT1
DMSO
5-Aza
PS
OS
PS
OS
DNMT1
DMSO
shDNMT1
PS
OS
PS
OS
CTGF
DMSO
5-Aza
PS
OS
PS
OS
CTGF
DMSO
shDNMT1
PS
OS
PS
OS
Cyclin A

Figure5a

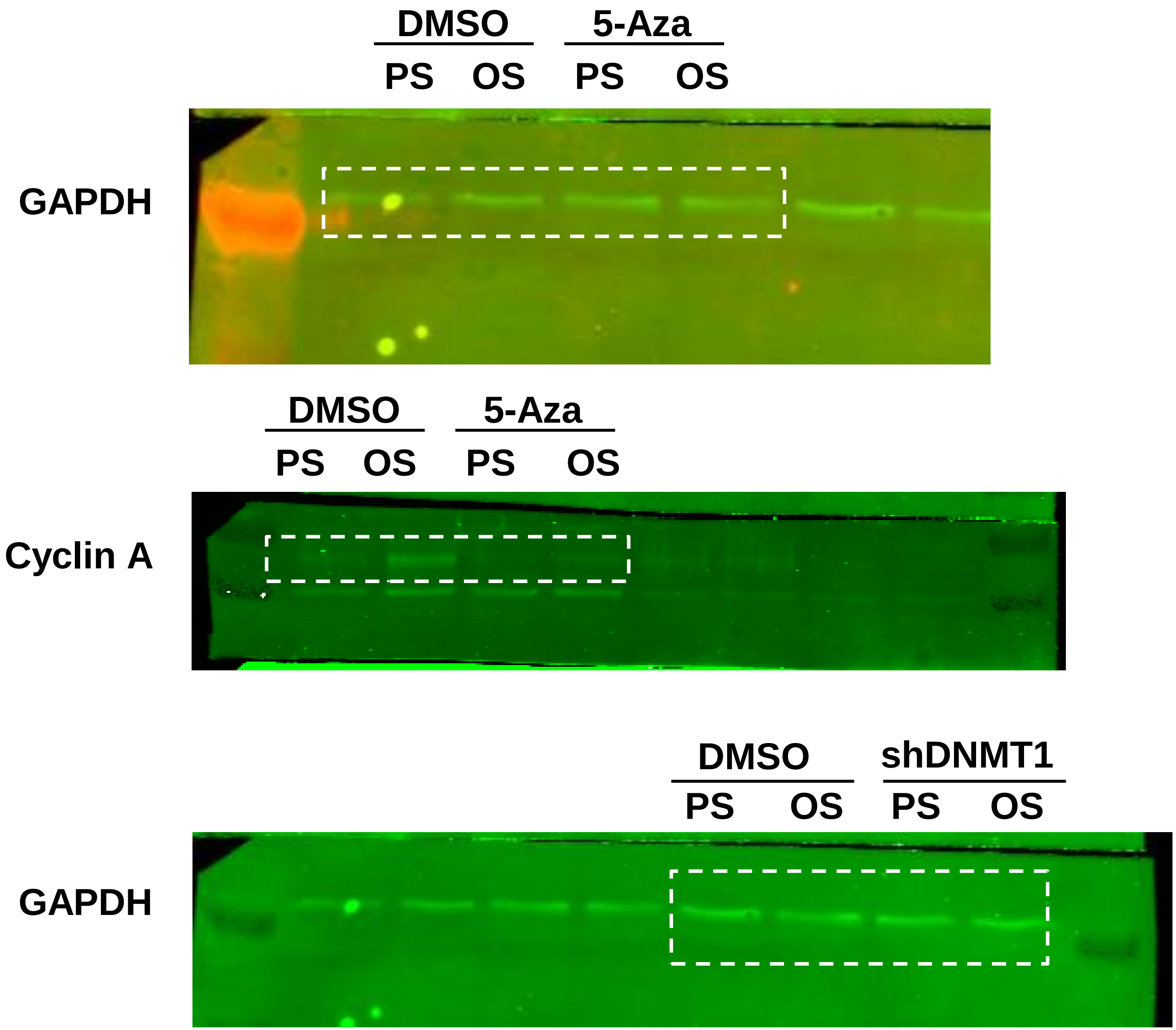
DMSO
5-Aza
PS
OS
PS
OS
GAPDH
DMSO
5-Aza
PS
OS
PS
OS
Cyclin A
DMSO
shDNMT1
PS
OS
PS
OS
GAPDH

Figure5a

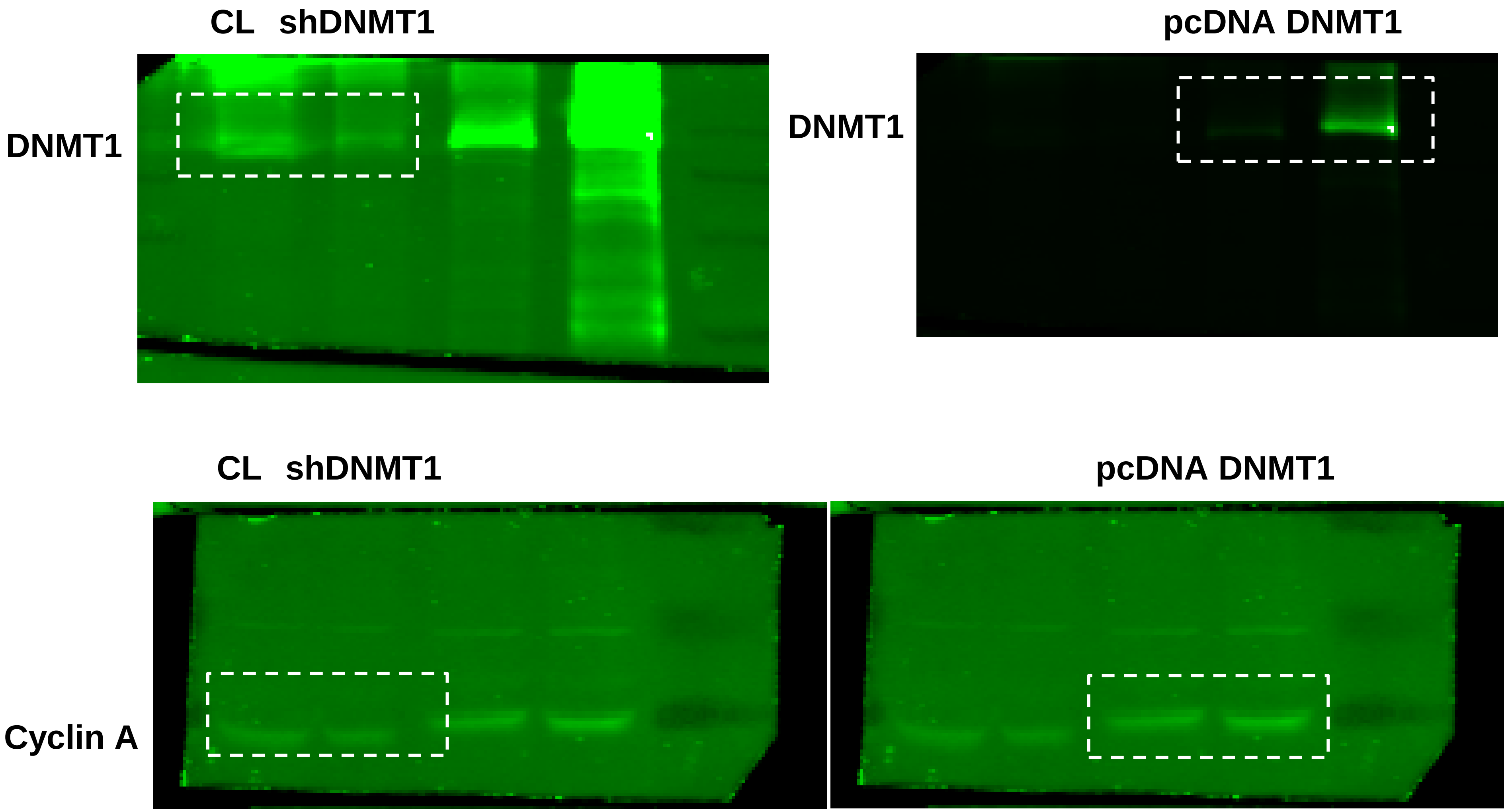
CL shDNMT1
pcDNA DNMT1
DNMT1
DNMT1
CL shDNMT1
pcDNA DNMT1
Cyclin A

Figure5e

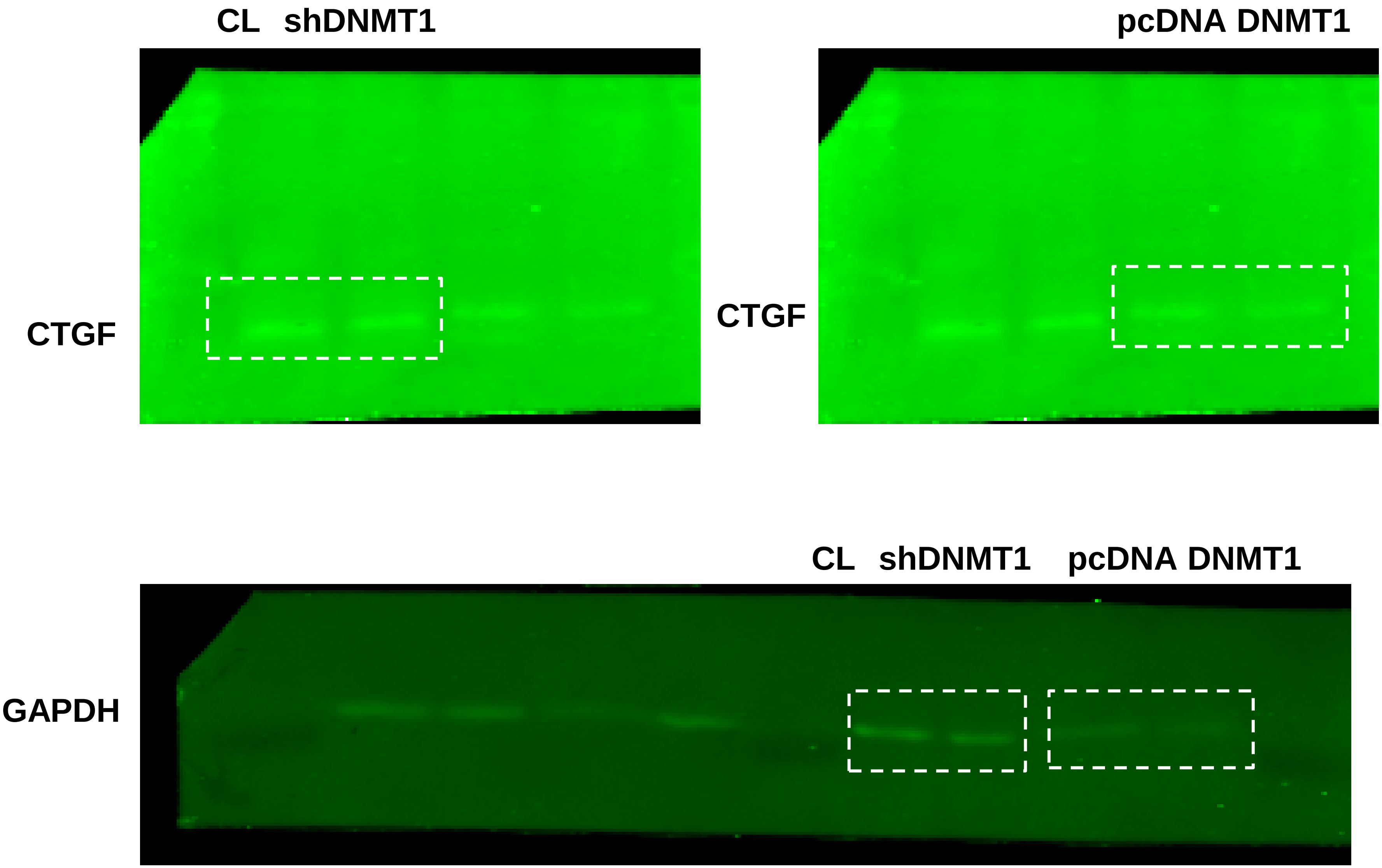

CL shDNMT1
pcDNA DNMT1
CTGF
CTGF
CL shDNMT1 pcDNA DNMT1
GAPDH

Figure5e

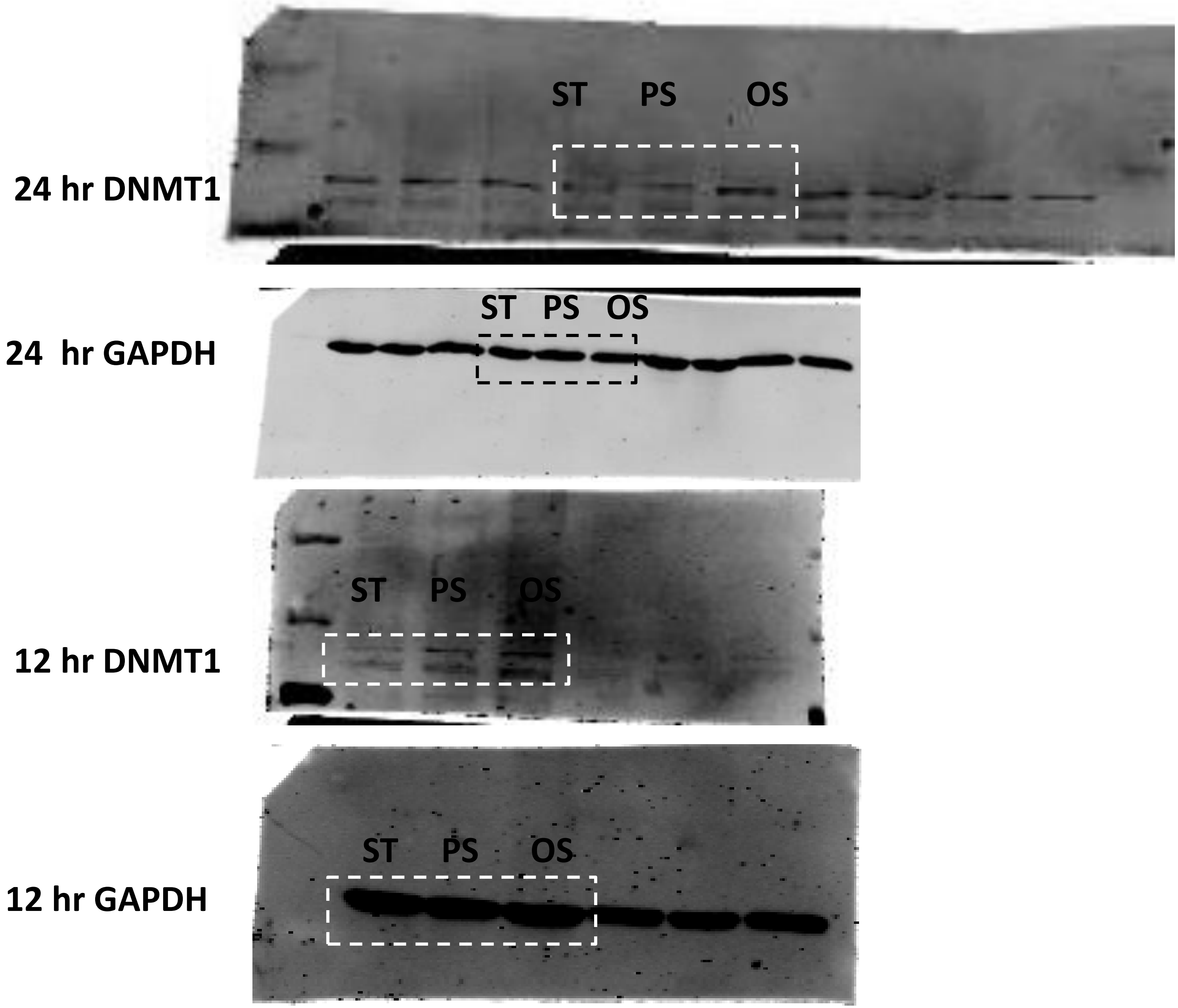
ST
PS
OS
24 hr DNMT1
ST
PS
OS
24 hr GAPDH
ST
PS
OS
12 hr DNMT1
ST
PS
OS
12 hr GAPDH

Figure S1
